# Effects of A1 Milk, A2 Milk and the Opioid-like Peptide β-Casomorphin-7 on the Proliferation of Human Peripheral Blood Mononuclear Cells

**DOI:** 10.3390/biom14060690

**Published:** 2024-06-13

**Authors:** Felix Gard, Lili M. Flad, Tanja Weißer, Hermann Ammer, Cornelia A. Deeg

**Affiliations:** 1 Chair of Animal Physiology, Department of Veterinary Sciences, Ludwig Maximilian University of Munich, D-82152 Martinsried, Germany; 2 Chair of Pharmacology, Toxicology and Pharmacy, Department of Veterinary Sciences, Ludwig Maximilian University of Munich, D-80539 Munich, Germany

**Keywords:** BCM-7, morphine, proliferation, PBMCs, CD4^+^ T cells, A1 milk, A2 milk

## Abstract

Special attention is given to cow’s milk and its variants, with ongoing discussions about health-related impacts primarily focusing on the A1 variant in contrast to the A2 variant. The difference between these variants lies in a single amino acid alteration at position 67 of β-casein. This alteration is presumed to make the A1 variant more susceptible to enzymatic breakdown during milk digestion, leading to an increased release of the peptide β-casomorphin-7 (BCM-7). BCM-7 is hypothesized to interact with µ-opioid receptors on immune cells in humans. Although BCM-7 has demonstrated both immunosuppressive and inflammatory effects, its direct impact on the immune system remains unclear. Thus, we examined the influence of A1 and A2 milk on Concanavalin A (ConA)-stimulated human peripheral blood mononuclear cells (PBMCs), as well as the effect of experimentally digested A1 and A2 milk, containing different amounts of free BCM-7 from β-casein cleavage. Additionally, we evaluated the effects of pure BCM-7 on the proliferation of ConA-stimulated PBMCs and purified CD4^+^ T cells. Milk fundamentally inhibited PBMC proliferation, independent of the β-casein variant. In contrast, experimentally digested milk of both variants and pure BCM-7 showed no influence on the proliferation of PBMCs or isolated CD4^+^ T cells. Our results indicate that milk exerts an anti-inflammatory effect on PBMCs, regardless of the A1 or A2 β-casein variant, which is nullified after in vitro digestion. Consequently, we deem BCM-7 unsuitable as a biomarker for food-induced inflammation.

## 1. Introduction

β-casein, constituting around 22% of the total protein in bovine milk [1], shows different phenotypes due to a genetic diversity among cattle [2]. There are currently known to be 17 different variants of β-casein, each differing in at least one amino acid [3]. Among the variants observed in European dairy cattle, the most prevalent are the A1 and A2 variants [4]. The ‘new world’ A1 variant differs from the ‘old world’ A2 variant, which is considered as the oldest form, in a mutation of a single amino acid at position 67 [5]. Histidine at position 67 in A1 β-casein renders the A1 β-casein more susceptible to enzymatic cleavage between position 66 and 67, a process less likely to occur in A2 β-casein, where proline resides at position 67 [6,7]. This enzymatic cleavage liberates a specific seven amino acid peptide known as β-casomorphin-7 (BCM-7). This peptide, though present in the sequence of β-casein in both the A1 and A2 variant, is released in higher quantities when digesting A1 milk compared to A2 milk [8]. This cleavage is conducted in three steps by pepsin, chymotrypsin, elastase and leucine aminopeptidase [9]. BCM-7, derived from bovine milk, was characterized for its opioid activity four decades ago [10]. Its initial three amino acids at the N-terminus of BCM-7 (Tyr-Pro-Phe) establish a structural similarity to endogenous peptides like enkephalins, endorphins or dynorphins, commencing with Tyr-Gly-Gly-Phe. Tyr at the N-terminus classifies it as an atypical opioid peptide [11]. After cleavage in the small intestine, this peptide is presumed to traverse the gut–blood barrier, potentially undergoing para- or transcellular transport through the intestinal brush border membrane (BBM) [12,13,14], particularly in conditions of heightened gut permeability, leading to elevated bloodstream concentrations [15]. BCM-7 has been detected in human plasma and urine post milk consumption [15,16,17]. A previous study showed that BCM-7 is degraded by dipeptidyl peptidase IV (DPP-IV) in vitro during transport in CaCo-2 cells [18]. In a more recent study, BCM-7 was cleaved by 79% after four hours of in vitro brush border membrane digestion (BBM-digestion), underscoring its limited bioavailability [19]. Various effects of BCM-7 are being discussed, including its ability to modulate the immune system through µ-opioid receptors [20], which are found on human immune cells, including T and B cells [21]. The impact of BCM-7 was described as exerting immunosuppressive effects on human lamina propria lymphocytes (LPLs) in vitro, a reaction that was blocked by naloxone, an opioid antagonist, thus confirming its opioid activity according to the authors of the respective study [22]. Conversely, BCM-7 was also reported to have an inflammatory effect on human PBMCs in vitro [23]. In another study, BCM-7 revealed both anti-inflammatory effects at lower concentrations and pro-inflammatory effects at higher concentrations on human PBMCs in vitro [24]. Additionally, hydrolysates from unidentified bovine β-casein variants have exhibited inhibitory effects on human PBMCs in vitro [25]. Consequently, studies comparing the effects of A1 and A2 milk as well as BCM-7 on the immune system remain inconclusive or contrary. Nonetheless, the A2 milk market is growing due to beneficial health claims about anti-inflammatory effects of A2 milk [26], although this remains a subject of debate and requires further investigation [27]. To date, a direct comparison evaluating a difference in the impact of A1 and A2 β-casein variants on primary human PBMCs has not been carried out. Therefore, our study aimed to explore the effect of A1 and A2 milk and their digestion products, particularly focusing on the heightened BCM-7 levels found in digested A1 milk, on the proliferation of human PBMCs. On top of that, we examined the isolated effect of pure BCM-7 on primary human PBMCs and its potential for modulating immune inflammation. This outcome was compared with the effect of morphine, serving as a positive control for the presumed opioid-like effects of BCM-7.

## 2. Materials and Methods

### 2.1. A1 and A2 Milk

A1 and A2 milk was used from three cows, each homozygous for either A1 or A2 β-casein. All cows were kept in the same herd and received the same diet. The cows were selected to represent three different lactation periods for each group and were of the same breed (Holstein Friesian). After sampling, the milk was cooled to 4 °C and initially skimmed four times using centrifugation (4 °C, 1000× *g*, 5 min). For use in the proliferation assay, the milks were pooled into an A1 and an A2 milk samples, respectively, and stored at −20 °C until use.

### 2.2. In Vitro Digestion of A1 and A2 Milk

For digestion, each milk was digested as previously described [28]. As a control for the effect of the digestion fluids, we also digested PBS (NaCl 136.9 mM, Na_2_HPO_4_ × 2H_2_O 8.1 mM, KH_2_PO_4_ 1.4 mM and KCl 2.6 mM; pH 7.4). Post-digestion, the proteolytic activity was stopped by using cOmplete EDTA-free protease inhibitor (Roche, Mannheim, Germany) followed by three cycles of freezing and thawing. cOmplete EDTA-free protease inhibitor (Roche) demonstrated anti-proliferative characteristics in previous preliminary in vitro proliferation assays when utilized at the manufacturer’s recommended concentration. Therefore, we opted to use one-tenth of the recommended quantity of the protease inhibitor. The digested milk was then pooled into an A1 and A2 digested milk pool and kept at −20 °C until usage.

### 2.3. Detection of BCM-7 in Digested Milk Using In-House Competitive ELISA

Since BCM-7 is generated during in vitro digestion of A1 but also of A2 milk [29] and ex vivo [8], we developed an in-house competitive ELISA using a mouse monoclonal BCM-7 antibody whose binding activity is not largely disturbed by milk components. This ELISA served as a control to confirm successful in vitro digestion and investigate whether BCM-7 was more prevalent in A1 milk compared to A2 milk after the digestion process. Standard curves were constructed with biotinylated BCM-7 (Peps4Life, Heidelberg, Germany) as a tracer. To exclude possible interference with the enzymes used for in vitro digestion of the milk, incubations were performed using digested PBS prepared as described above. By using digested PBS, we ensured that the digestion process did not interfere with the measured quantity of BCM-7 in the digested milk, particularly in terms of interactions with the antibody binding sites in the ELISA. The digested milk was initially diluted at a ratio of 1:10 in PBS for the assay. Considering the 1:4 dilution in the digestion fluids, this resulted in a total dilution of 1:40. The optical density was then determined with a microplate reader at 450 nm (Tecan, Crailsheim, Germany), and the data underwent quantitative analysis using freely available GainData ELISA Calculator software (https://www.arigobio.com/elisa-analysis; accessed on 8 December 2023; Arigo, Hsinchu, Taiwan).

### 2.4. Isolation of PBMCs from Human Blood Samples

Venous blood samples, comprising 40 mL each from 15 voluntary donors, were provided in sodium-heparin-coated tubes and diluted at a ratio of 1:2 in PBS (pH 7.4). After density gradient centrifugation (room temperature, 500× *g*, 25 min) using Pancoll separating solution (PanBiotech, Aidenbach, Germany), PBMCs were collected from the buffy coat and washed twice in PBS (pH 7.4). Only donors tolerant to milk were asked to provide blood samples for this study. 

### 2.5. In Vitro Cell Proliferation

After resuspension in RPMI 1640 (PanBiotech) with 1% penicillin–streptomycin (P/S) (PanBiotech) and 10% fetal calf serum (FCS) (Sigma-Aldrich, Darmstadt, Germany), 2 × 10^5^ PBMCs were immediately seeded into each well of 96-well U-bottom plates (Sarstedt, Nümbrecht, Germany) and stimulated in triplicates with 1 µg/mL Concanavalin A (ConA) (Sigma-Aldrich). One triplicate of each PBMC per donor was stimulated only with ConA to serve as the control for additional treatments (ConA control). One triplicate of each PBMC per donor was not stimulated with ConA and was incubated only in medium (RPMI 1640 with 1% P/S and 10% FCS) to serve as a control for ConA-induced proliferation (negative control). To evaluate the effect of milk on the proliferation of PBMCs, we added unprocessed or digested milk in a final concentration of 1% to further triplicates of ConA-stimulated PBMCs. Since previous investigations exhibited a decrease in proliferation caused by the digestion fluids, which contain enzymes, taurocholic acid, and protease inhibitors, we had to set up a proper control for digested milk to nullify this anti-proliferative effect. Therefore, we used digested PBS in the same final concentration of 1% (ConA digest control). To assess a possible opioidergic effect, ConA-stimulated PBMCs were subjected to a dilution series of morphine sulfate (Sigma-Aldrich), ranging from 1 nM to 100 µM, with each dilution step being tenfold, including an additional dilution point at 63 µM. The same dilution series was applied to BCM-7 (Peps4Life, Heidelberg, Germany). The concentration of 63 µM is equivalent to 50 µg/mL of BCM-7, a dilution that has been employed in prior studies [22]. The dilution range from 1 nM to 100 µM was chosen based on previous research indicating BCM-7’s contradictory inflammatory [23] and anti-inflammatory [24] effects on cell proliferation in lower concentrations and inflammatory [23,24] and anti-inflammatory [22] effects in higher concentrations. Eight hours before harvesting, PBMCs were labeled with 1 µCi [^3^H]-thymidine (PerkinElmer, Rodgau, Germany) per well. Thymidine is utilized by cells to replicate DNA and marks newly synthesized DNA [30]. After a 48 h stimulation period, PBMCs were harvested on MicroBeta filter mats (PerkinElmer) with a FilterMate harvester (PerkinElmer). Radioactivity of each well was quantified using a β-scintillation counter MicroBeta^2^ (PerkinElmer), presenting measurements in counts per minute (cpm). The degree of cellular uptake of [^3^H]-thymidine is indicative of cells undergoing DNA replication in the S-phase of the cell cycle and therefore represents cell proliferation [31]. 

### 2.6. Separation of CD4^+^ T Cells by Magnetic-Activated Cell Sorting (MACS)

To assess the influence of BCM-7 on CD4^+^ T cells, we isolated CD4^+^ T cells from PBMCs via MACS. A total of 5 × 10^7^ PBMCs were suspended in 3 mL of MACS buffer (PBS with 0.5% BSA and 2 mM EDTA, pH 7.2) and incubated with 1 µg/mL of mouse mab to human CD4 (clone RPA-T4, Bio-Rad, Feldkirchen, Germany) for 20 min at 4 °C. Subsequently, PBMCs were washed in MACS buffer. In the next step, PBMCs were resuspended in 80 µL of MACS buffer, and 20 µL of anti-mouse-IgG1 MicroBeads (Miltenyi Biotec, Bergisch Gladbach, Germany) was added. After 15 min of incubation, PBMCs were washed and resuspended in 500 µL of MACS buffer. PBMCs were then applied onto LS columns (Miltenyi Biotec) and magnetic separation was performed on a QuadroMACS separator (Miltenyi Biotec). After rinsing with 9 mL of MACS buffer, the flow-through was discarded. The LS column was then removed from the separator, and the CD4^+^ T cells were flushed out by adding 5 mL of MACS buffer and firmly applying a plunger (Miltenyi Biotec). The purity of the CD4^+^ T cell fraction was analyzed using flow cytometry (Quanteon, Agilent, Waldbronn, Germany) and ranged between 92% and 98%. In the proliferation assay, the CD4^+^ T cells were stimulated with ConA and 50 µg/mL (63 µM) BMC-7 and compared to ConA-stimulated CD4^+^ T cells (ConA control for CD4^+^ T cells). One triplicate of each CD4^+^ T cell sample per donor was not stimulated with ConA and was incubated only in RPMI 1640 with 1% P/S and 10% FCS to serve as a control for ConA-induced proliferation (negative control for CD4^+^ T cells).

### 2.7. Statistical Analysis

Proliferation was quantified in counts per minute (cpm), and the mean cpm values of all triplicates were normalized by dividing them by the mean values of the respective ConA control triplicates of each 96-well plate and donor. The control conditions for incubation in ConA and milk, morphine sulfate, or BCM-7 consisted of incubation only in ConA, while the control condition for incubation in ConA with digested milk consisted of incubation in ConA with PBS subjected to the identical digestion protocol. As a result, the ConA control values were standardized to a value of one, ensuring more accurate inter-donor comparisons. Outliers with a z-score greater than two were excluded. To analyze the resulting indices, we conducted a Kruskal–Wallis test followed by Dunn’s post hoc test to compare each group. To perform these analyses, we utilized R version 2023.09.1 (Posit PBC, Boston, MA, USA) along with the ‘rstatics’ (version 0.7.2) and ‘tidyverse’ (version 2.0.0) packages. Visualization was carried out using Prism version 5.04 (GraphPad, Boston, MA, USA).

## 3. Results

### 3.1. ConA-Induced Proliferation of PBMCs

To verify if the proliferation of PBMCs was induced by ConA, we compared the proliferation indices of PBMCs or CD4^+^ T cells only incubated in medium (negative control) with those of ConA-stimulated PBMCs or CD4^+^ T cells (ConA control). Since we used ConA-stimulated cells as a control and all proliferation values were divided by these control values, the resulting proliferation index of the ConA control itself was calculated at 1 for each PBMC per individual donor. 

The stimulation index (SI) of the PBMC negative control was calculated at 0.04 ± 0.04 SD, indicating a mean 25-fold higher stimulation of the ConA control (SI = 1) compared to the negative control. This observation demonstrates the successful induction of proliferation in PBMCs by ConA. The stimulation index of the CD4^+^ T cell negative control was calculated at 0.03 ± 0.04 SD, which results in a mean 33-fold higher stimulation of CD4^+^ T cells after incubation with ConA (SI = 1). The higher proliferation rate of ConA-stimulated CD4^+^ T cells (33-fold) compared to ConA-stimulated PBMCs (25-fold) demonstrates the proliferative effect of ConA on T cells.

As previously described, the control conditions for ConA-stimulation with digested milk consisted of ConA-stimulation with digested PBS (ConA digest control). To evaluate whether proliferation was induced by ConA in the ConA digest control, we compared the ConA digest control in medium-incubated PBMCs (negative control). The stimulation index of the negative control compared to the ConA digest control was calculated at 0.12 ± 0.17 SD. This resulted in a mean eight-fold higher proliferation of the ConA digest control (SI = 1) compared to the negative control. Reduced proliferation of the digest control (eight-fold) compared to the ConA control (25-fold) demonstrates that digestion fluids inhibit proliferation and proper controls for digested substances are necessary.

### 3.2. A1 and A2 Milk Inhibited Proliferation of PBMCs

**Figure 1:**
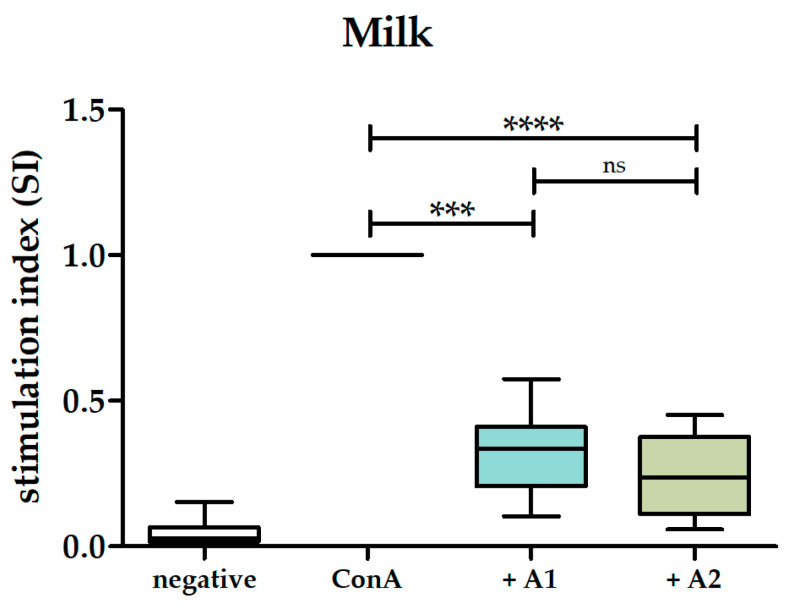
Effects of A1 and A2 milk on ConA-induced proliferation of human PBMCs. Box and whisker plots represent stimulation indices of ConA-stimulated PBMCs following incubation in 1% A1 (light blue) and 1% A2 (light green) milk, n = 10. ConA-stimulated PBMCs served as control and were set to 1 (black line), n = 10. PBMCs incubated only in medium (negative control) served as the control for ConA-induced proliferation (white), n = 10. Non-parametric Kruskal–Wallis test followed by Dunn’s post hoc test was performed to test for statistical significance; ns, *p* > 0.05; ***, *p* ≤ 0.001; ****, *p* ≤ 0.0001.

To evaluate whether the A1 and A2 variants of β-casein in milk exhibit any effect on the ConA-induced proliferation of PBMCs, we first investigated the impact of A1 and A2 milk on the proliferation of ConA-stimulated PBMCs. A1 milk exhibited a significant mean inhibition of 68% on the proliferation of ConA-stimulated PBMCs (stimulation index of 0.32 ± 0.14 SD). Similarly, A2 milk significantly inhibited the proliferation of ConA-stimulated PBMCs by an average of 75% (stimulation index of 0.25 ± 0.14 SD). No statistically significant difference was detected when comparing the anti-proliferative effects of A1 milk and A2 milk (Figure 1).

### 3.3. Digested A1 and A2 Milk Contained BCM-7 and Did Not Alter the Proliferation of PBMCs

**Figure 2:**
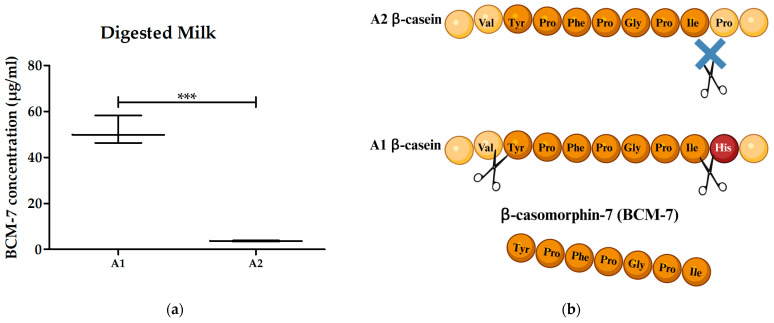
(**a**) BCM-7 concentration in A1 and A2 milk after in vitro digestion. BCM-7 concentration in A1 milk (n = 3) was 14-fold higher than in the A2 milk (n = 3) after in vitro digestion. Student’s *t*-test was performed to test for statistical significance; ***, *p* ≤ 0.001. (**b**) Presumed proteolytic cleavage sites in bovine β-casein. Amino acid alteration at position 67 in A1 β-casein from proline to histidine renders it more susceptible to enzymatic cleavage and leads to a higher release of BCM-7 compared to the A2 variant. The blue X indicates reduced enzymatic cleavage between the amino acids isoleucine and proline in A2 β-casein compared to A1 β-casein.

Since no difference between the effect of A1 and A2 milk on the proliferation of ConA-stimulated PBMCs was observed, we investigated whether digestion of A1 and A2 milk might have an impact through the release of BCM-7 and further breakdown products. By doing so, we also investigated whether BCM-7 is more abundant in the A1 milk compared to A2 milk after in vitro digestion. Each digested milk sample underwent testing for its BCM-7 content using our in-house ELISA. The mean amount of BCM-7 in digested milk was determined to be 14-fold higher (51.5 ± SD 6.1 µg/mL) in digested A1 milk compared to digested A2 milk (3.7 ± SD 0.2 µg/mL) (Figure 2a). 

**Figure 3:**
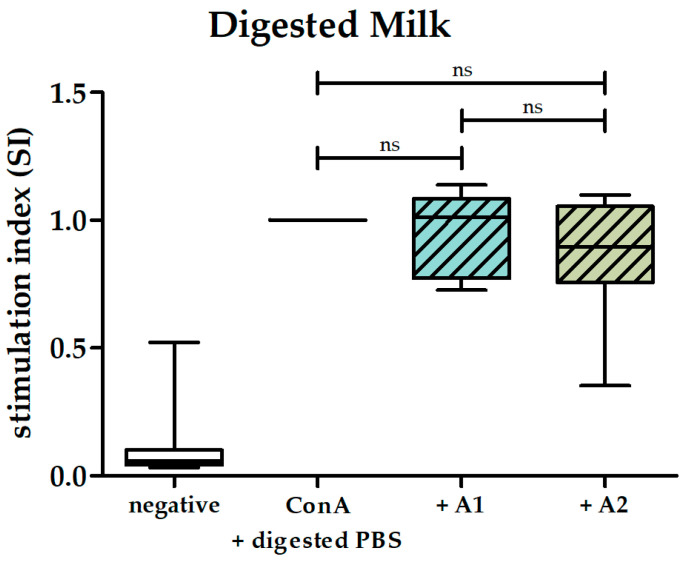
Effects of digested A1 and A2 milk on ConA-induced proliferation of human PBMCs. Box and whisker plots represent stimulation indices of ConA-stimulated PBMCs following incubation in 1% digested A1 (striped light blue) and 1% digested A2 (striped light green) milk, n = 10. ConA-stimulated PBMCs incubated in 1% digested PBS served as control and were set to 1 (black line), n = 10. PBMCs incubated only in medium (negative control) served as the control for ConA-induced proliferation (white), n = 10. Non-parametric Kruskal–Wallis test followed by Dunn’s post hoc test was performed to test for statistical significance; ns, *p* > 0.05.

Considering 1% pooled digested milk usage in the proliferation assay, the calculated BCM-7 concentration in the assay was 5.2 µg/mL for the digested A1 milk pool and 0.4 µg/mL for the digested A2 milk pool. Despite the considerable 13-fold variance in BCM-7 amounts, digested A1 milk had no effect on the proliferation of ConA-stimulated PBMCs, resulting in a 5% mean reduction in stimulation (stimulation index of 0.95 ± 0.15 SD). The stimulation index for digested A2 milk was lower (0.78 ± 0.23 SD). However, no statistical significance was observed. Furthermore, no statistically significant difference was evident between digested A1 milk and digested A2 milk (Figure 3).

### 3.4. BCM-7 at Several Concentrations Did Not Alter the Proliferation of PBMCs and CD4^+^ T Cells

**Figure 4:**
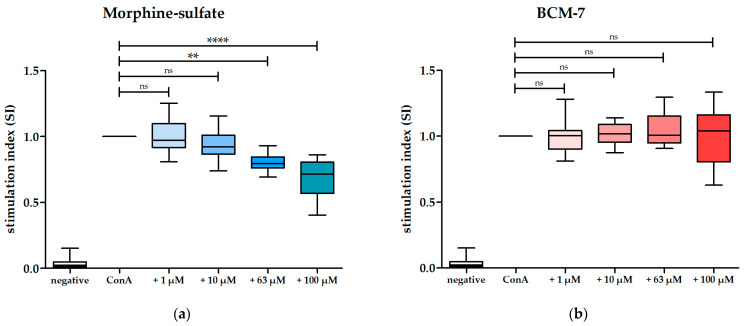
(**a**) Effect of morphine sulfate on ConA-stimulated PBMCs. Box and whisker plots represent stimulation indices of ConA-stimulated PBMCs following incubation in morphine sulfate in various concentrations (from light to dark blue), n = 15. ConA-stimulated PBMCs served as a control and were set to 1 (black line), n = 15. PBMCs incubated only in medium (negative control) served as the control for ConA-induced proliferation (white), n = 15. Non-parametric Kruskal–Wallis test followed by Dunn’s post hoc test was performed to test for statistical significance; ns, *p* > 0.05; **, *p* ≤ 0.01; ****, *p* ≤ 0.0001. (**b**) Effect of BCM-7 on ConA-stimulated PBMCs. Box and whisker plots represent stimulation indices of ConA-stimulated PBMCs following incubation in BCM-7 in various concentrations (from light to dark red), n = 15. ConA-stimulated PBMCs served as control and were set to 1 (black line), n = 15. PBMCs incubated only in medium (negative control) served as the control for ConA-induced proliferation (white), n = 15. Non-parametric Kruskal–Wallis test followed by Dunn’s post hoc test was performed to test for statistical significance; ns, *p* > 0.05.

Given that digested milk did not affect ConA-induced PBMC proliferation, despite the higher abundance of BCM-7 in digested A1 milk, our subsequent investigation focused on the impact of pure BCM-7 on the proliferation of ConA-stimulated PBMCs. This approach aimed to eliminate potentially disturbing matrix effects of milk and its components. As BCM-7 is hypothesized to act through binding to the µ-opioid receptor [23], we first inspected whether the proliferation of ConA-stimulated PBMCs is affected by the µ-opioid agonist morphine sulfate under the conditions of our assay. The expected inhibition of proliferation by morphine sulfate [32] was observed starting from a concentration of 63 µM. At lower concentrations, no statistically significant difference compared to the control was observed. This demonstrated that opioids are able to inhibit ConA-induced PBMC proliferation in our assay and may serve as a positive control (Figure 4a; Supplementary Figure S36). We further investigated the impact of BCM-7 using the same concentrations. No statistically significant differences in proliferation were observed between ConA-stimulated PBMCs and ConA-stimulated PBMCs incubated with various concentrations of BCM-7 (Figure 4b; Supplementary Figure S37).

**Figure 5:**
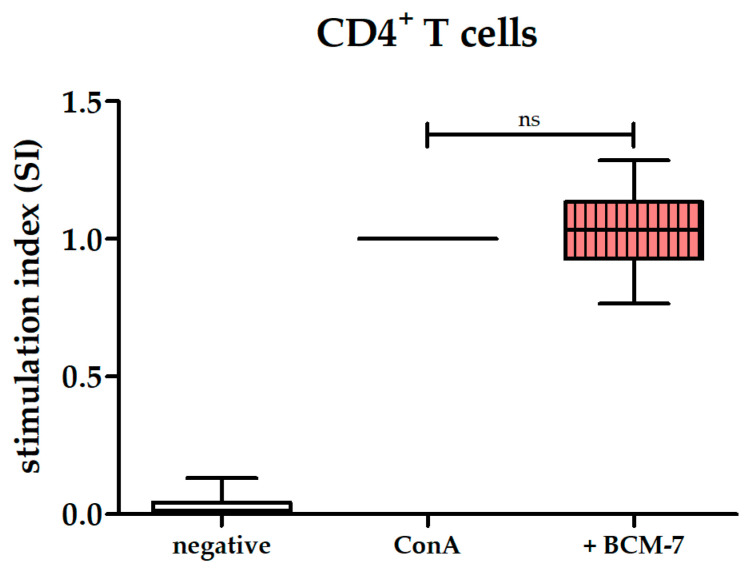
Effect of BCM-7 on CD4^+^ T cells. Box and whisker plot represents stimulation index of ConA-stimulated CD4^+^ T cells following incubation in 63 µM BCM-7 (light red, vertically striped), n = 10. ConA-stimulated CD4^+^ T cells served as control and were set to 1 (black line), n = 10. CD4^+^ T cells incubated only in medium (negative control for CD4^+^ T cells) served as the control for ConA-induced proliferation (white), n = 10. Non-parametric Kruskal–Wallis test followed by Dunn’s post hoc test was performed to test for statistical significance; ns, *p* > 0.05.

T cells are the primary cell fraction activated by ConA [33]. Given that BCM-7 demonstrated no effect on the ConA-induced proliferation of PBMCs, we subsequently investigated whether pure CD4^+^ T cells would respond to BCM-7, since effects of BCM-7 may be masked by the various cell types in PBMCs. However, BCM-7 showed no significant effect on ConA-induced proliferation of CD4^+^ T cells (Figure 5).

## 4. Discussion

BCM-7, which is generated in increased amounts from A1 β-casein during digestion [8], is being discussed as a potential marker for negative effects on the immune system [16,34]. However, currently, there is a lack of data to assess the impact of BCM-7 and β-casein variants on immune cells [35]. Therefore, the aim of this study was to gather information on the effects of BCM-7 as well as A1 and A2 milk on human PBMCs. In doing so, we were able to demonstrate that milk in general exhibited anti-inflammatory effects on ConA-stimulated human PBMCs, but there was no significant difference between the milk containing either the A1 or A2 β-casein variant. To our knowledge, no prior study has directly compared the effects of the two β-casein variants in milk on human ConA-stimulated PBMCs.

Nevertheless, previous studies have examined the impact of milk on PBMC proliferation without specifically considering the distinct β-casein variant [36,37]. For example, a prior study investigated the influence of milk and its components on lipopolysaccharide (LPS)-stimulated human PBMCs in vitro, disregarding the specific β-casein variant present [36]. Their findings revealed increased levels of Interleukin-10 (IL-10) in PBMC supernatants after incubation with milk or pure casein [36]. IL-10, known to be produced by leukocytes [38], inhibits mitogen-induced T cell proliferation [39] and exerts inhibitory effects on various immune cells, such as B cells, limiting the overall immune response [40]. The observed antiproliferative effect of milk in our study may be linked to the induction of IL-10 production in PBMCs by milk or casein. However, we did not investigate the specific mechanism responsible for inhibiting PBMC stimulation. Notably, our study used ConA as the stimulating mitogen, while the previously mentioned study applied LPS to induce PBMC proliferation [36]. This suggests that the mechanism governing milk’s inhibitory properties on immune cell proliferation might differ depending on the stimulant used, and further investigation is essential to fully understand said mechanism in ConA-stimulated human PBMCs. 

Another study investigated the effects of caseins on the proliferation of mouse spleen lymphocytes and rabbit Peyer’s patch cells after ConA-, phytohemagglutinin (PHA)-, pokeweed mitogen (PWM)- and LPS-induced stimulation in vitro [37]. Notably, only κ-casein was associated with an anti-proliferative effect, not α_s1_- or β-casein [37]. While these findings hint at a potential association between κ-casein and the observed anti-inflammatory response in our study, it is crucial to note that we have not specifically examined the effects of individual caseins (α, β, κ) on human PBMCs. Therefore, we cannot confirm the anti-proliferative effect of κ-casein. Further investigations are necessary to validate whether these results can be extended to human PBMCs as well as to reveal which protein in milk is responsible for the inhibition of ConA-induced proliferation of human PBMCs and through which mechanism this inhibition occurs. 

In addition to caseins, whey proteins, comprising the second major fraction of proteins in milk [41], have also been suggested to potentially modulate inflammation in periphery [42]. However, in concentrations similar to those used in our study, whey proteins did not display any effects on the ConA-induced proliferation of human PBMCs in vitro [43]. This strongly suggests that whey proteins may not be accountable for the observed anti-proliferative effect noted in our research. Nevertheless, our findings clearly demonstrate that milk with different β-casein variants does not exhibit significant differences in influencing the proliferation of ConA-stimulated human PBMCs in vitro.

The concentration of 1% milk used in our study is theoretically equivalent to a total uptake of 50 mL of milk in the bloodstream of a 70 kg adult with an estimated total blood volume of five liters. It is important to mention that the probability of milk components interacting with PBMCs in concentrations used in our study is less likely under typical healthy in vivo conditions due to the high digestibility of milk proteins [44] and the blood–gut barrier primarily absorbing amino acids and small peptides [45]. However, consumption of buffering foods like milk can neutralize gastric acidity [46], resulting in reduced pepsin activities and potential passage of intact milk proteins into the small intestine [47]. Before undergoing further breakdown by intestinal enzymes [45], proteins can potentially be absorbed and transported transcellularly by membranous epithelial cells [48,49], eventually being released at the basolateral side of the enterocytes [50]. Therefore, the interaction between milk and PBMCs remains physiologically plausible [51], especially in scenarios of heightened gut permeability, where it is conceivable that larger peptides and proteins might cross the gut–blood barrier in even higher concentrations [52]. BCM-7, a peptide believed to exert opioid-like effects on immune cells [24], was reported to be released during digestion of milk [53]. To examine this phenomenon, we subjected both A1 and A2 milk to an in vitro digestion process and measured their respective BCM-7 contents. In our study, this process generated 51.5 µg/mL of BCM-7 in A1 milk and 3.7 µg/mL in A2 milk. Previous studies reported lower concentrations in A1 milk and lower or undetectable concentrations in A2 milk after ex vivo [8] or in vitro digestion [7,9]. The observed variations in BCM-7 concentrations among studies could be attributed to the use of different digestion protocols, encompassing varying enzymes, concentrations, and incubation periods. In our research, the use of a standardized method with a longer incubation period, representing physiological conditions [28], allowed us to detect the highest amount of BCM-7 among these studies. Ensuring consistency in the digestion process is vital for accurate comparisons between the amount of BCM-7 in digested milk samples. While the absolute values varied, increased BCM-7 release during the digestion of A1 milk compared to A2 milk was consistently demonstrated, not only in our studies but also by other experts in the field [6,7,8,9,29,54,55]. These findings support the hypothesis that the mutation of A1 β-casein at position 67 leads to elevated BCM-7 release via enzymatic cleavage [9]. Theoretically, one liter of milk containing 10 g of β-casein [41] could yield a maximum of 400 µM or 315 µg/mL of BCM-7. However, the actual release of BCM-7 from milk after digestion was notably lower than the theoretically possible release, as observed in our study and in the studies conducted by other groups [6,7,8,9,29,54,55,56]. The observed disparity between the detected and theoretically expected amounts of BCM-7 after digestion implies that not every β-casein molecule is cleaved at the positions required for BCM-7 generation. Consequently, the actual emergence of BCM-7 during milk digestion may be lower than theoretically predicted.

The potential impact of the BCM-7 from digested A1 and A2 milk on the proliferation of ConA-stimulated PBMCs was further examined in our study. Surprisingly, post-digestion of both A1 and A2 milk, we observed the disappearance of any significant anti-inflammatory effects on human PBMCs. This suggests that the active agents responsible for the anti-inflammatory properties in milk were inactivated during in vitro digestion. Furthermore, despite observing a 13-fold higher release of BCM-7 after digestion in A1 milk compared to A2 milk, our study revealed no significant difference in the effect on proliferation of human PBMCs between the two milk variants containing either the A1 or A2 β-casein. This demonstrates that the divergent BCM release from the two β-casein variants has no discernible impact on the proliferation of human PBMCs. To our knowledge, we are the first to directly compare the effects of digested A1 and A2 milk variants on the ConA-induced proliferation of human PBMCs in vitro.

A previous study investigated the impact of digested milk on human PBMC proliferation but did not consider its β-casein variant [25]. In this study, digested milk, with unknown β-casein variants, was reported to inhibit PHA-induced proliferation of human PBMCs in vitro [25]. While the mechanism behind the observed inhibition remains undisclosed in said study [25], our findings contradict their report of digested milk inhibiting PBMC proliferation induced by PHA [25]. The use of pepsin and trypsin for milk digestion [25] might result in an incomplete breakdown of milk components, affecting the quantity of bioactive milk molecules. In contrast, our standardized digestion method, representing gastric and jejunal phases [28], likely enabled a more comprehensive breakdown. This extensive digestion may have cleaved bioactive molecules responsible for the observed inhibitory effect in milk, potentially nullifying its initial impact on PBMC proliferation. Nevertheless, we clearly showed that the variation of β-casein in milk did not differ in its impact on the proliferation of human ConA-stimulated PBMCs in vitro, even after digestion. 

BCM-7, linked to opioid activity [10], was previously presumed to demonstrate anti-proliferative effects on stimulated human immune cells [22]. Although our study confirmed the significant presence of BCM-7 particularly in digested A1 milk, our investigation revealed no discernible impact of digested milk on ConA-stimulated human PBMCs. This led us to conclude that BCM-7 in digested milk does not affect human PBMCs as anticipated. To confirm that the observed absence of any effect was not influenced by potential interferences introduced during digestion, we took additional measures to assess the specific impact of synthetically generated pure BCM-7 on the proliferation of ConA-stimulated PBMCs. A previous study demonstrated an inhibitory effect of synthetically generated BCM-7 at 10 µg/mL (12.6 µM) after a five-day incubation period with ConA-stimulated lamina propria lymphocytes (LPLs) in vitro [22]. From this, the authors inferred that this indicated opioid activity of BCM-7. Interestingly, in the initial 48 h, no effect of BCM-7 on the primary lymphocytes of the ileal and colonic mucosa was observed, aligning with our results in PBMCs. The much-discussed effect of BCM-7 only surfaced after five days. This timeframe is highly unusual for polyclonal cell stimulation and may lead to the cessation of cell stimulation by ConA [57]. By using positive controls with morphine in our assays, our study clearly demonstrated that opioid receptors on PBMCs develop a significant dose-dependent response within just 48 h. These anti-inflammatory effects of morphine occurred at concentrations of 63 µM. However, under the same conditions, BCM-7 showed no significant effect on the proliferation of PBMCs, questioning its postulated effects on inflammation and its role as a potential marker for food-induced inflammation. 

In contrast to our findings demonstrating no influence of BCM-7 on the proliferation of ConA-stimulated PBMCs, a previous study observed modulating effects of BCM-7 on cell proliferation [24]. This study noted a pro-inflammatory effect at a concentration of 10 µM and an anti-inflammatory effect at 1 nM BCM-7 on ConA-stimulated human PBMCs after 72 h in vitro [24]. The authors attributed these outcomes to BCM-7’s opioid activity but did not substantiate any opioid effects through positive controls in their assay [24]. Our study, on the other hand, clearly demonstrated the anti-inflammatory impact of opioids on human ConA-stimulated PBMCs, while BCM-7 exhibited no discernible effects on PBMCs, indicating that it does not function as an opioid. 

In another study, BCM-7, tested at concentrations ranging from 1 fM to 10 µM, was reported to increase the PHA-L-induced proliferation of human PBMCs in vitro [23]. Contrarily, opioids used in said study revealed anti-proliferative effects on PBMCs during PHA-L-stimulation, aligning with our results, showing that BCM-7 did not act via opioid receptors [23]. The exact mechanism of BCM-7 enhancing proliferation on PHA-L-stimulated PBMCs was not revealed by the authors [23]. While our study utilized ConA to induce proliferation via CD28 and herpes virus entry mediator (HVEM) pathways [58], their proliferation was induced by PHA-L, stimulating T cells via toll-like receptor 4 (TLR4) [59]. The reported impact of BCM-7 on PBMCs stimulated through TLR4 [23] indicates a potential interaction pathway for BCM-7. Additional investigation with various cell stimulants is essential to delineate the specific interaction pathways of BCM-7 with human PBMCs.

Given the diverse cell subtypes within PBMCs and their heterogeneous response to different types of stimuli, it is possible that the detection of potential reactivity to BCM-7 in certain subsets might be masked. Stimulants such as PHA-L primarily stimulate T cells, particularly CD4^+^ T cells, through TLR4 activation [60]. Considering that BCM-7 has previously exhibited stimulatory effects when using PHA-L on PBMCs [23], we conducted further investigations specifically focusing on the CD4^+^ T cell subset, which plays a pivotal role in immune regulation [42]. However, BCM-7 did not have any significant effect on ConA-stimulated CD4^+^ T cells, indicating that CD4^+^ T cells are not a target population for BCM-7. Further investigations are needed to reveal potential effects on further PBMC subtypes.

For BCM-7 to potentially induce any effects on PBMCs, absorption by the gut is necessary. Assuming the ingestion of one liter of A1 milk, releasing a similar quantity of BCM-7 during in vivo digestion as observed in our in vitro digestion experiments (51.5 µg/mL), and a 100% absorption rate via the BBM without degradation, the theoretical quantity of absorbed BCM-7 is approximately 51.5 mg. Diluted within the bloodstream of a 70 kg adult with an estimated total of 5 L of blood, this would equate to a concentration of 10.3 µg/mL or 13 µM BCM-7. Consequently, the range of BCM-7 used in our study, from 1 nM to 100 µM, corresponds to a consumption equivalent of 77 µL up to 7.7 L of A1 milk. Considering BCM-7’s physiologically limited bioavailability [19], which results in lower concentrations in the blood stream, it might be impossible to achieve such high blood concentrations through the consumption of milk. Our study shows that BCM-7, even in higher concentrations than theoretically physiologically achievable, exhibited no effect on human PBMC and CD4^+^ T cell subset in vitro. 

Preconceived notions about the alleged negative effects of BCM-7 on health have often been used to disseminate unquestioned claims about A1 milk [61]. A2 milk consumption, on the other hand, was proposed to lead to higher levels of the anti-oxidant glutathione [62] in blood compared to A1 milk consumption [26]. These findings were utilized to advocate for the superiority of A2 milk due to higher glutathione (GSH) levels after A2 milk consumption, while neglecting to acknowledge that A1 milk also increased GSH levels in blood [26]. Despite this, the consumption of both A1 and A2 milk led to slightly increased BCM-7 levels in blood plasma [26]. This suggests that differences in GSH levels are most likely not attributed to BCM-7. The a2 Milk Company, a sponsor of studies in this field [5,26], has utilized these assertions to promote its product despite inconclusive results [63]. In an expanding market, it is crucial to meticulously examine study findings and consider the entire product when discussing health impacts. 

## 5. Conclusions

Milk exhibits an anti-inflammatory effect on human PBMCs in vitro. This effect is independent of the A1 or A2 β-casein variant and ceases when milk is digested. Digestion of milk results in the release of different amounts of BCM-7, depending on the A1 or A2 β-casein variant. In our experiments, neither BCM-7 from milk digestion nor synthetic BCM-7 had an impact on the activation status of human PBMCs. Thus, the previously described opioid effects of BCM-7 could not be confirmed, and we conclude that BCM-7 is not useful as a biomarker for food-induced inflammation. The specific protein responsible for the anti-inflammatory effect of undigested milk on PBMCs remains unidentified. Therefore, further investigations are essential for clarification and identification of this protein. 

## Data Availability

All raw data are presented in Supplementary Figures S1–S35, respectively. The data from the results section presented in this study are available in the article or in Supplementary Figures S36 and S37.

## Supplementary Materials

The following supporting information can be downloaded at https://www.mdpi.com/article/10.3390/biom14060690/s1: Figures S1–S10: Effects of A1 and A2 milk and digested A1 and A2 milk on ConA-stimulated human PBMCs of each individual donor 1–10; Figures S11–S25: Effects of morphine sulfate and BCM-7 on ConA-stimulated human PBMCs of each individual donor 1–15; Figures S26–S35: Effect of BCM-7 on ConA-stimulated CD4+ T cells of each individual donor 1–10; Figure S36: Effect of morphine sulfate on ConA-stimulated PBMCs; Figure S37: Effect of BCM-7 on ConA-stimulated PBMCs.
