# Supplementary material for: Effects of A1 Milk, A2 Milk and the Opioid-like Peptide β-Casomorphin-7 on the Proliferation of Human Peripheral Blood Mononuclear Cells
Source: Biomolecules. 2024 Jun 13;14(6):690. doi: 10.3390/biom14060690 (PMC11201611; doi:10.3390/biom14060690)
Supplement: Supplementary file 1 [file biomolecules-14-00690-s001.zip › biomolecules-2790143-supplementary.pdf]

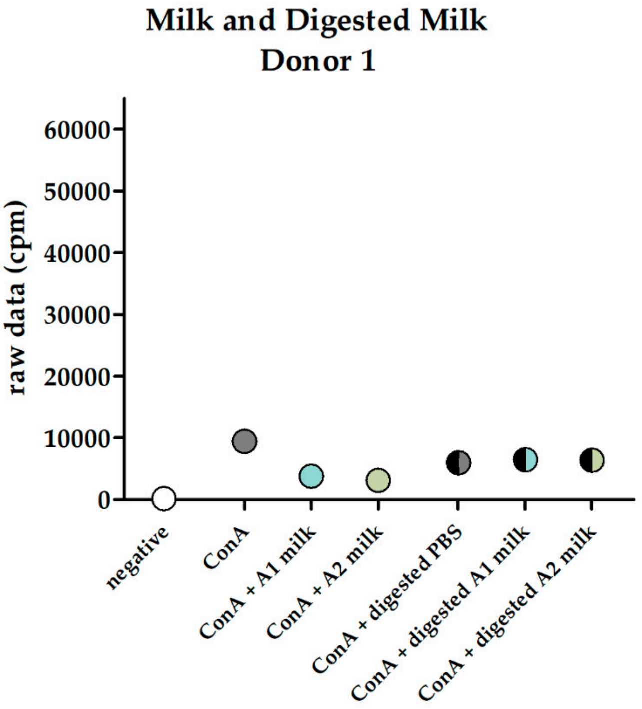

Supplementary Figure S1: Effects of A1 and A2 milk and digested A1 and A2 milk on ConA-stimulated human PBMC of donor 1. Scatter plots represent raw count per minute (cpm)-values of ConA-stimulated PBMC following incubation in 1% A1 milk (light blue), 1% A2 milk (light green), 1% digested A1 milk (striped light blue) and 1% digested A2 milk (striped light green). ConA-stimulated PBMC served as a control for ConA + 1% milk stimulation (grey). ConA-stimulated PBMC incubated in 1% digested PBS served as control for ConA + 1% digested milk stimulation (striped grey). PBMC incubated only in medium (negative control) served as the control for ConA-induced proliferation (negative, white).

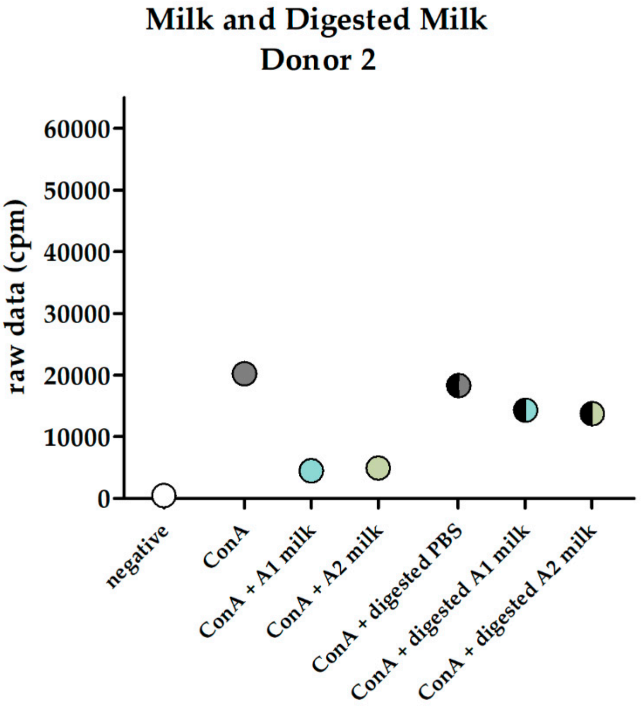

Supplementary Figure S2: Effects of A1 and A2 milk and digested A1 and A2 milk on ConA-stimulated human PBMC of donor 2. Scatter plots represent raw cpm-values of ConA-stimulated PBMC following incubation in 1% A1 milk (light blue), 1% A2 milk (light green), 1% digested A1 milk (striped light blue) and 1% digested A2 milk (striped light green). ConA-stimulated PBMC served as a control for ConA + 1% milk stimulation (grey). ConA-stimulated PBMC incubated in 1% digested PBS served as control for ConA + 1% digested milk stimulation (striped grey). PBMC incubated only in medium (negative control) served as the control for ConA-induced proliferation (negative, white).

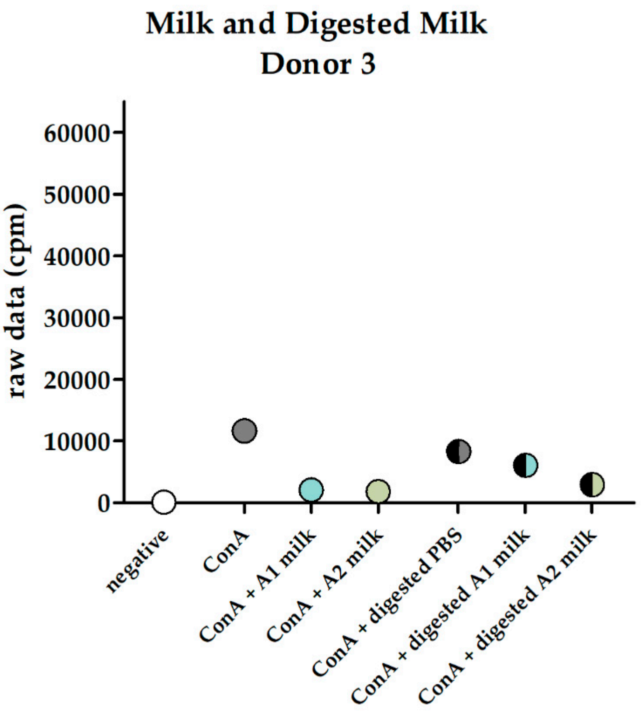

Supplementary Figure S3: Effects of A1 and A2 milk and digested A1 and A2 milk on ConA-stimulated human PBMC of donor 3. Scatter plots represent raw cpm-values of ConA-stimulated PBMC following incubation in 1% A1 milk (light blue), 1% A2 milk (light green), 1% digested A1 milk (striped light blue) and 1% digested A2 milk (striped light green). ConA-stimulated PBMC served as a control for ConA + 1% milk stimulation (grey). ConA-stimulated PBMC incubated in 1% digested PBS served as control for ConA + 1% digested milk stimulation (striped grey). PBMC incubated only in medium (negative control) served as the control for ConA-induced proliferation (negative, white).

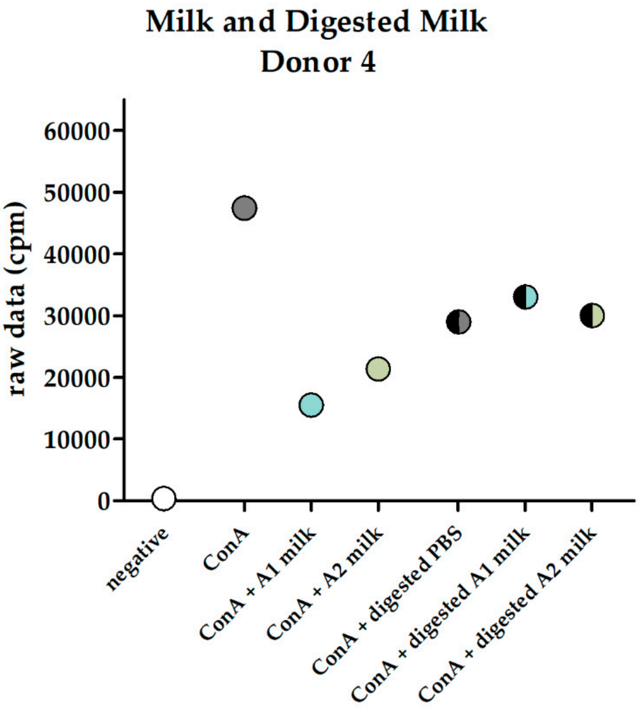

Supplementary Figure S4: Effects of A1 and A2 milk and digested A1 and A2 milk on ConA-stimulated human PBMC of donor 4. Scatter plots represent raw cpm-values of ConA-stimulated PBMC following incubation in 1% A1 milk (light blue), 1% A2 milk (light green), 1% digested A1 milk (striped light blue) and 1% digested A2 milk (striped light green). ConA-stimulated PBMC served as a control for ConA + 1% milk stimulation (grey). ConA-stimulated PBMC incubated in 1% digested PBS served as control for ConA + 1% digested milk stimulation (striped

grey). PBMC incubated only in medium (negative control) served as the control for ConA-induced proliferation (negative, white).

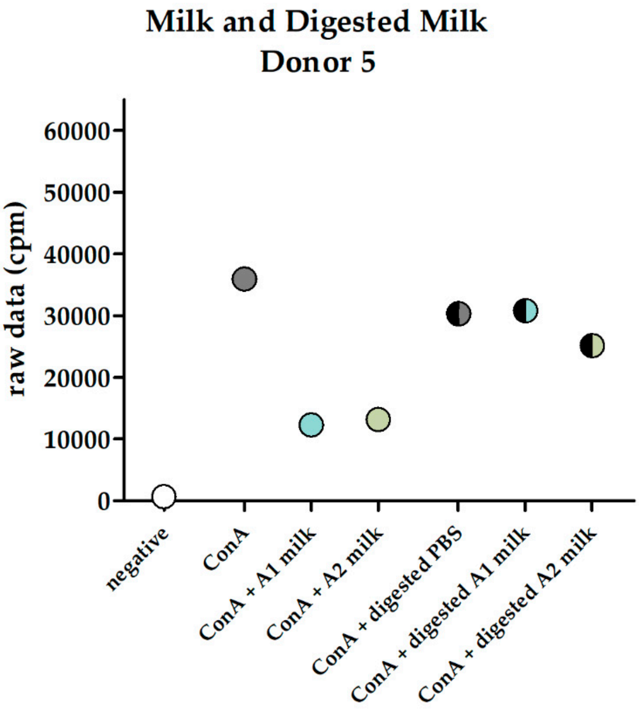

Supplementary Figure S5: Effects of A1 and A2 milk and digested A1 and A2 milk on ConA-stimulated human PBMC of donor 5. Scatter plots represent raw cpm-values of ConA-stimulated PBMC following incubation in 1% A1 milk (light blue), 1% A2 milk (light green), 1% digested A1 milk (striped light blue) and 1% digested A2 milk (striped light green). ConA-stimulated PBMC served as a control for ConA + 1% milk stimulation (grey). ConA-stimulated PBMC incubated in 1% digested PBS served as control for ConA + 1% digested milk stimulation (striped grey). PBMC incubated only in medium (negative control) served as the control for ConA-induced proliferation (negative, white).

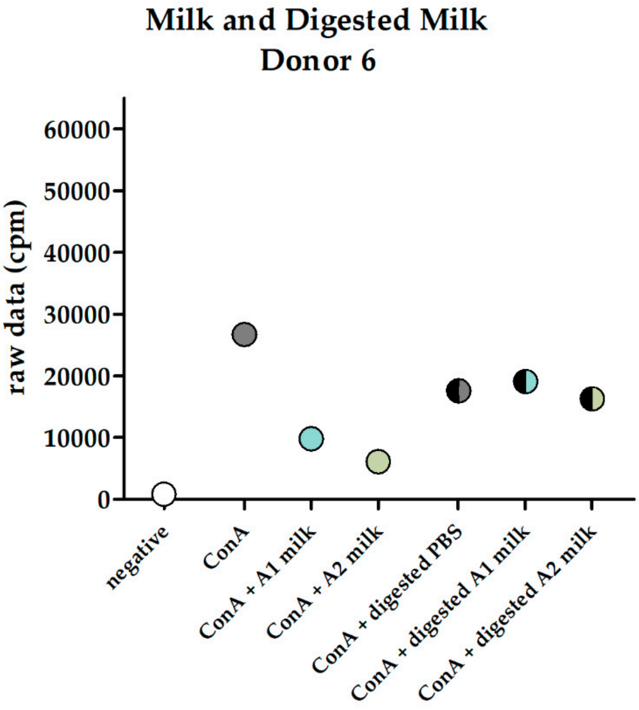

Supplementary Figure S6: Effects of A1 and A2 milk and digested A1 and A2 milk on ConA-stimulated human PBMC of donor 6. Scatter plots represent raw cpm-values of ConA-stimulated PBMC following incubation in 1% A1 milk (light blue), 1% A2 milk (light green), 1% digested A1 milk (striped light blue) and 1% digested A2 milk

(striped light green). ConA-stimulated PBMC served as a control for ConA + 1% milk stimulation (grey). ConA-stimulated PBMC incubated in 1% digested PBS served as control for ConA + 1% digested milk stimulation (striped grey). PBMC incubated only in medium (negative control) served as the control for ConA-induced proliferation (negative, white).

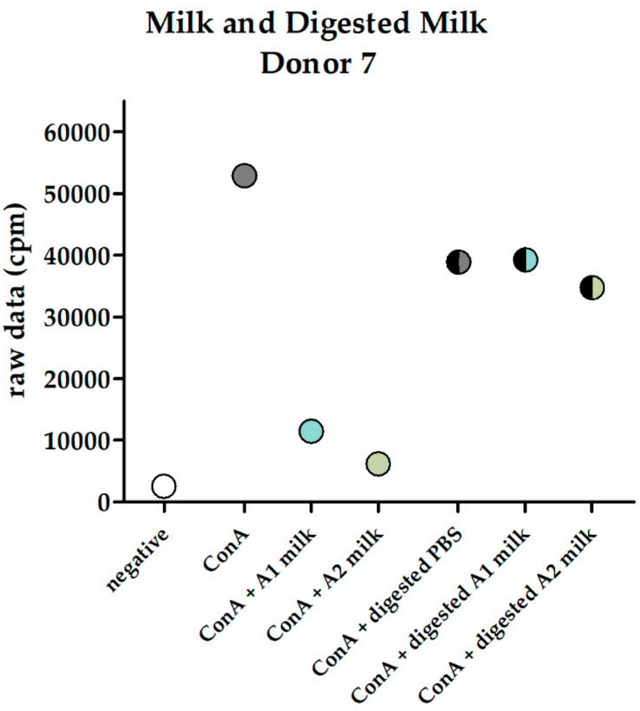

Supplementary Figure S7: Effects of A1 and A2 milk and digested A1 and A2 milk on ConA-stimulated human PBMC of donor 7. Scatter plots represent raw cpm-values of ConA-stimulated PBMC following incubation in 1% A1 milk (light blue), 1% A2 milk (light green), 1% digested A1 milk (striped light blue) and 1% digested A2 milk (striped light green). ConA-stimulated PBMC served as a control for ConA + 1% milk stimulation (grey). ConA-stimulated PBMC incubated in 1% digested PBS served as control for ConA + 1% digested milk stimulation (striped grey). PBMC incubated only in medium (negative control) served as the control for ConA-induced proliferation (negative, white).

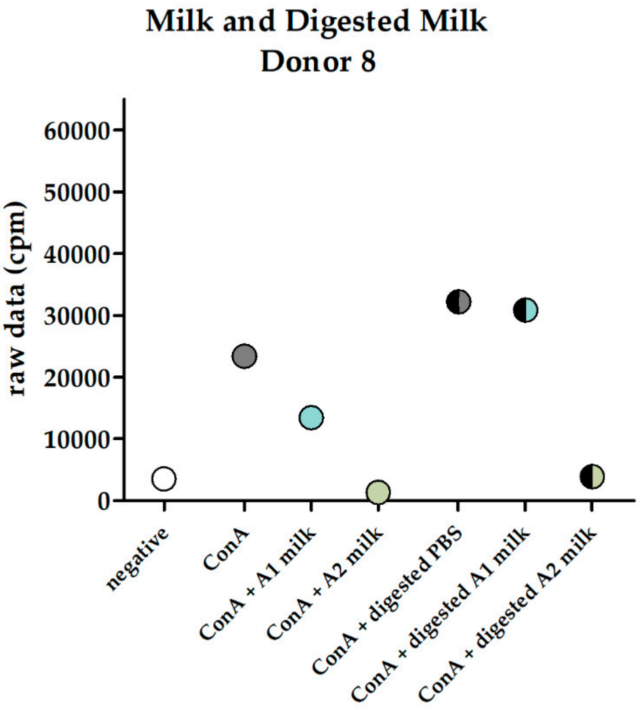

Supplementary Figure S8: Effects of A1 and A2 milk and digested A1 and A2 milk on ConA-stimulated human PBMC of donor 8. Scatter plots represent raw cpm-values of ConA-stimulated PBMC following incubation in 1% A1 milk (light blue), 1% A2 milk (light green), 1% digested A1 milk (striped light blue) and 1% digested A2 milk (striped light green). ConA-stimulated PBMC served as a control for ConA + 1% milk stimulation (grey). ConA-stimulated PBMC incubated in 1% digested PBS served as control for ConA + 1% digested milk stimulation (striped grey). PBMC incubated only in medium (negative control) served as the control for ConA-induced proliferation (negative, white).

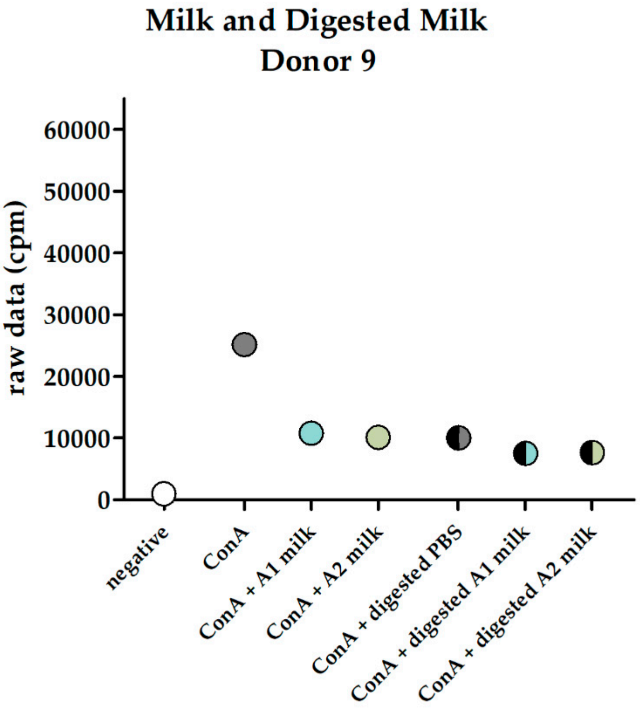

Supplementary Figure S9: Effects of A1 and A2 milk and digested A1 and A2 milk on ConA-stimulated human PBMC of donor 9. Scatter plots represent raw cpm-values of ConA-stimulated PBMC following incubation in 1% A1 milk (light blue), 1% A2 milk (light green), 1% digested A1 milk (striped light blue) and 1% digested A2 milk (striped light green). ConA-stimulated PBMC served as a control for ConA + 1% milk stimulation (grey). ConA-stimulated PBMC incubated in 1% digested PBS served as control for ConA + 1% digested milk stimulation (striped grey). PBMC incubated only in medium (negative control) served as the control for ConA-induced proliferation (negative, white).

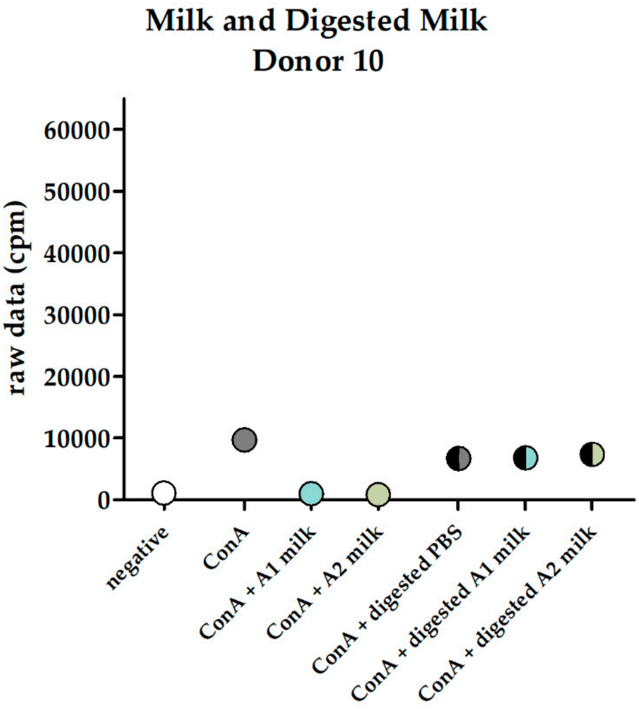

Supplementary Figure S10: Effects of A1 and A2 milk and digested A1 and A2 milk on ConA-stimulated human PBMC of donor 10. Scatter plots represent raw cpm-values of ConA-stimulated PBMC following incubation in 1% A1 milk (light blue), 1% A2 milk (light green), 1% digested A1 milk (striped light blue) and 1% digested A2 milk (striped light green). ConA-stimulated PBMC served as a control for ConA + 1% milk stimulation (grey). ConA-stimulated PBMC incubated in 1% digested PBS served as control for ConA + 1% digested milk stimulation (striped grey). PBMC incubated only in medium (negative control) served as the control for ConA-induced proliferation (negative, white).

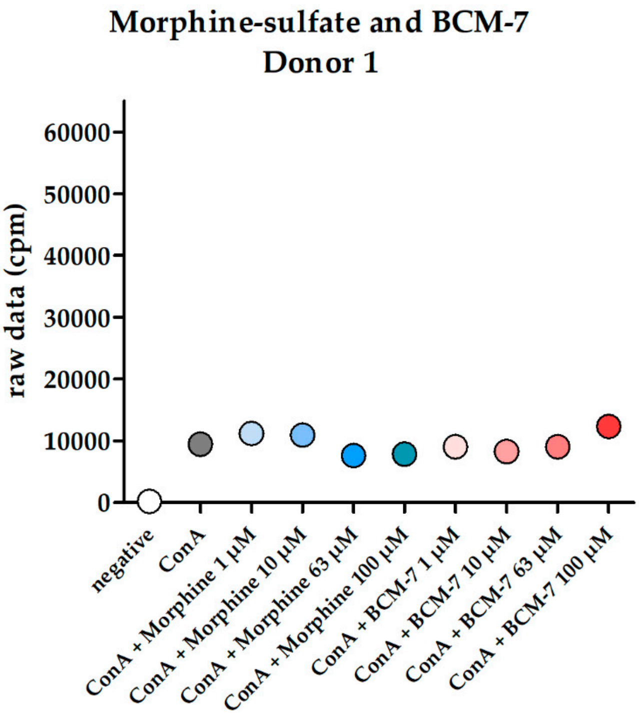

Supplementary Figure S11: Effects of morphine-sulfate and BCM-7 on ConA-stimulated human PBMC of donor 1. Scatter plots represent raw cpm-values of ConA-stimulated PBMC following incubation in morphine-sulfate (from light to dark blue) and BCM-7 (from light to dark red) in various concentrations. ConA-stimulated PBMC served as control (grey). PBMC incubated only in medium (negative control) served as the control for ConA-induced proliferation (white).

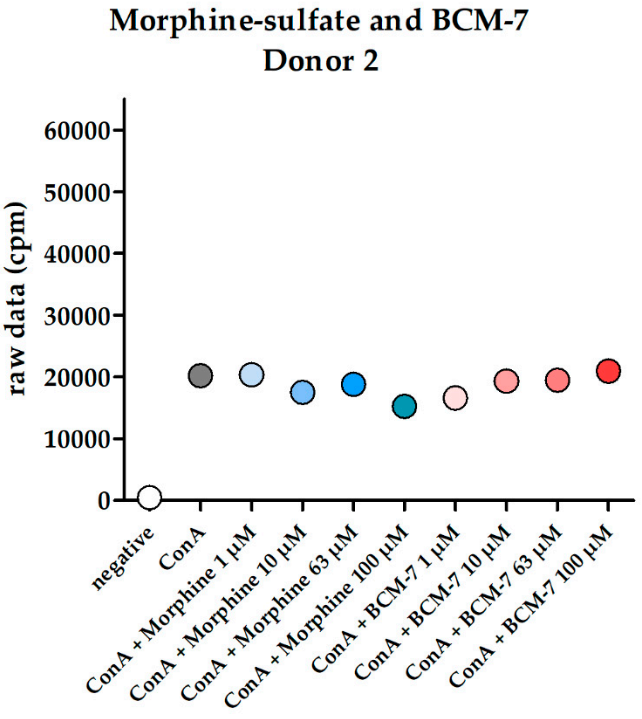

Supplementary Figure S12: Effects of morphine-sulfate and BCM-7 on ConA-stimulated human PBMC of donor 2. Scatter plots represent raw cpm-values of ConA-stimulated PBMC following incubation in morphine-sulfate (from light to dark blue) and BCM-7 (from light to dark red) in various concentrations. ConA-stimulated PBMC served as control (grey). PBMC incubated only in medium (negative control) served as the control for ConA-induced proliferation (white).

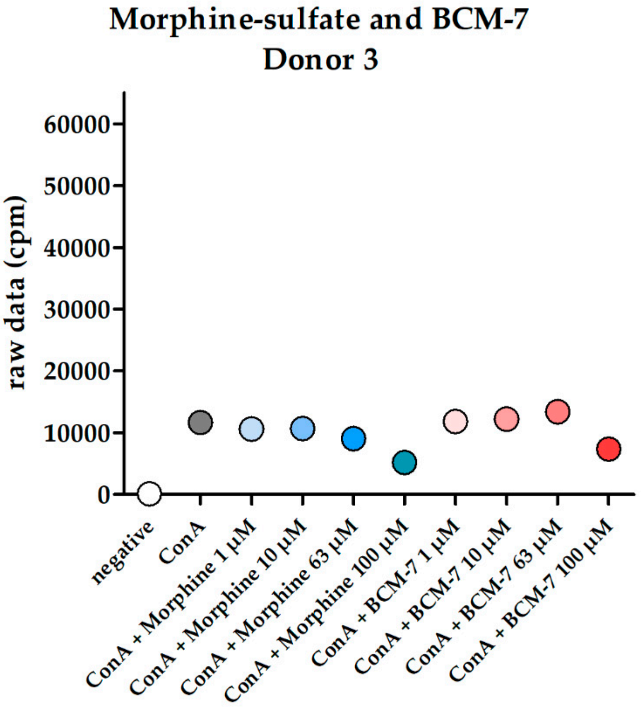

Supplementary Figure S13: Effects of morphine-sulfate and BCM-7 on ConA-stimulated human PBMC of donor 3. Scatter plots represent raw cpm-values of ConA-stimulated PBMC following incubation in morphine-sulfate (from light to dark blue) and BCM-7 (from light to dark red) in various concentrations. ConA-stimulated PBMC served as control (grey). PBMC incubated only in medium (negative control) served as the control for ConA-induced proliferation (white).

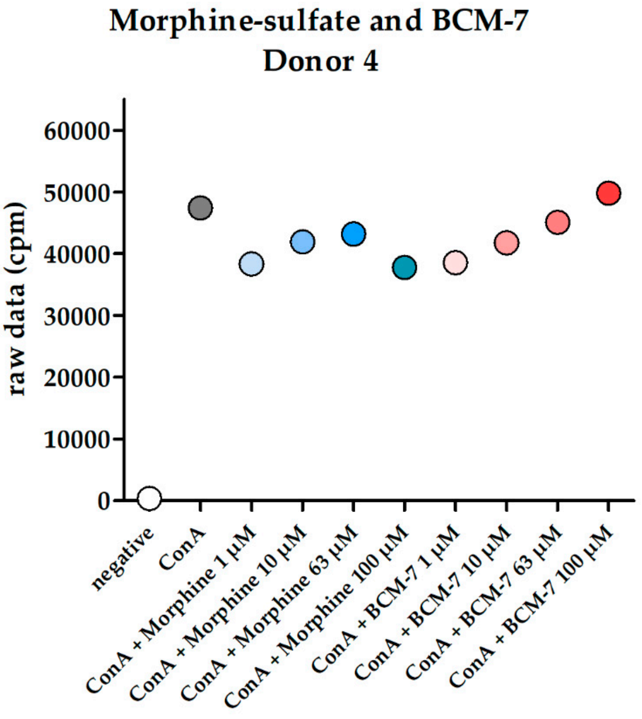

Supplementary Figure S14: Effects of morphine-sulfate and BCM-7 on ConA-stimulated human PBMC of donor 4. Scatter plots represent raw cpm-values of ConA-stimulated PBMC following incubation in morphine-sulfate (from light to dark blue) and BCM-7 (from light to dark red) in various concentrations. ConA-stimulated PBMC served as control (grey). PBMC incubated only in medium (negative control) served as the control for ConA-induced proliferation (white).

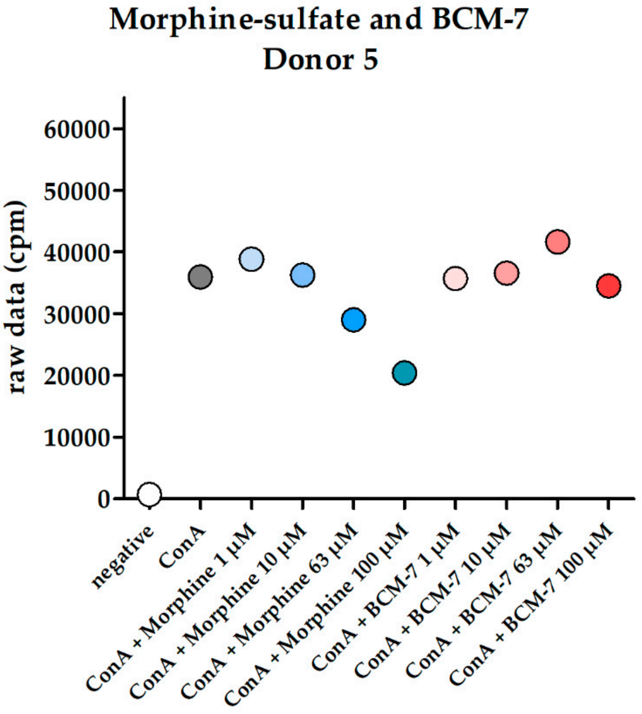

Supplementary Figure S15: Effects of morphine-sulfate and BCM-7 on ConA-stimulated human PBMC of donor 5. Scatter plots represent raw cpm-values of ConA-stimulated PBMC following incubation in morphine-sulfate (from light to dark blue) and BCM-7 (from light to dark red) in various concentrations. ConA-stimulated PBMC served as control (grey). PBMC incubated only in medium (negative control) served as the control for ConA-induced proliferation (white).

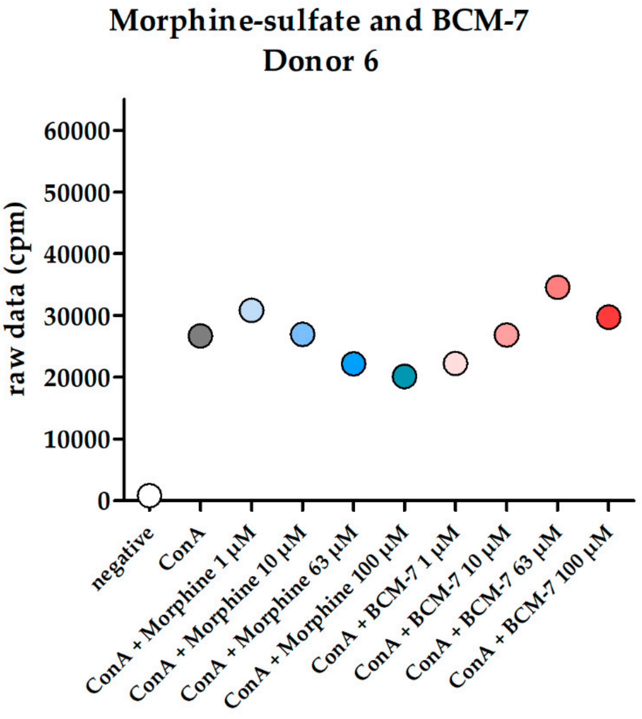

Supplementary Figure S16: Effects of morphine-sulfate and BCM-7 on ConA-stimulated human PBMC of donor 6. Scatter plots represent raw cpm-values of ConA-stimulated PBMC following incubation in morphine-sulfate (from light to dark blue) and BCM-7 (from light to dark red) in various concentrations. ConA-stimulated PBMC served as control (grey). PBMC incubated only in medium (negative control) served as the control for ConA-induced proliferation (white).

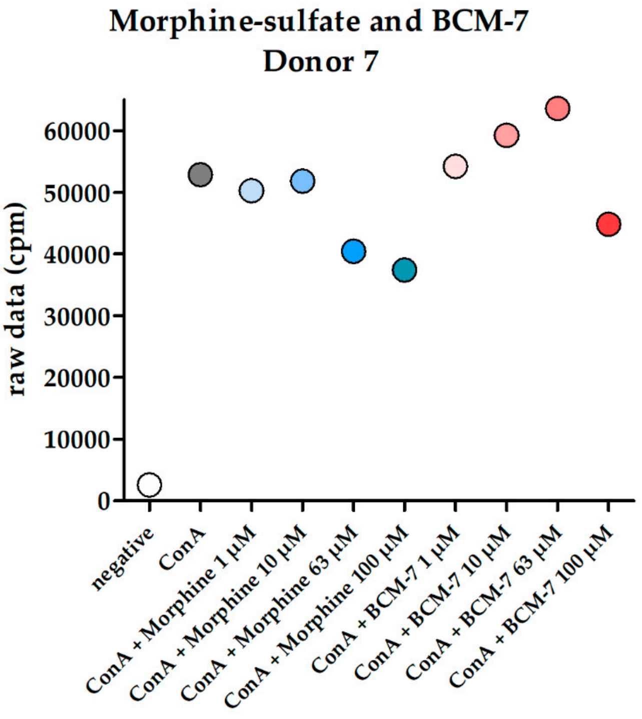

Supplementary Figure S17: Effects of morphine-sulfate and BCM-7 on ConA-stimulated human PBMC of donor 7. Scatter plots represent raw cpm-values of ConA-stimulated PBMC following incubation in morphine-sulfate (from light to dark blue) and BCM-7 (from light to dark red) in various concentrations. ConA-stimulated PBMC served as control (grey). PBMC incubated only in medium (negative control) served as the control for ConA-induced proliferation (white).

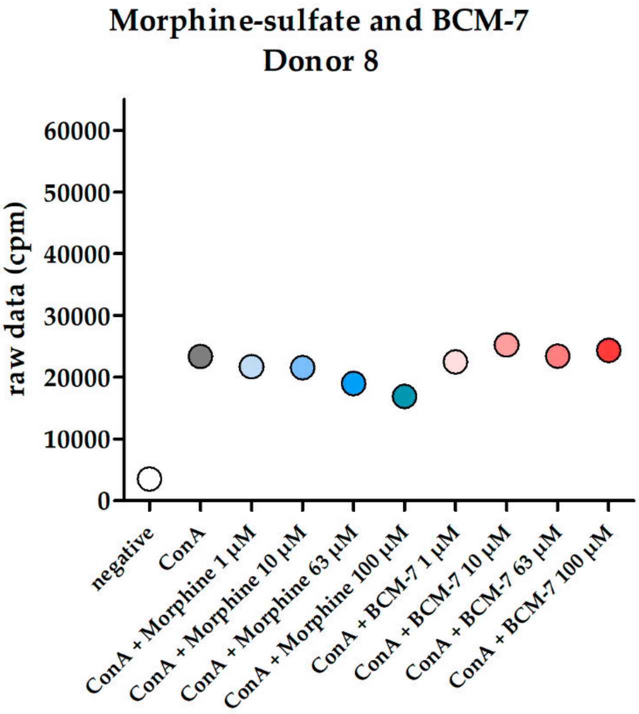

Supplementary Figure S18: Effects of morphine-sulfate and BCM-7 on ConA-stimulated human PBMC of donor 8. Scatter plots represent raw cpm-values of ConA-stimulated PBMC following incubation in morphine-sulfate (from light to dark blue) and BCM-7 (from light to dark red) in various concentrations. ConA-stimulated PBMC served as control (grey). PBMC incubated only in medium (negative control) served as the control for ConA-induced proliferation (white).

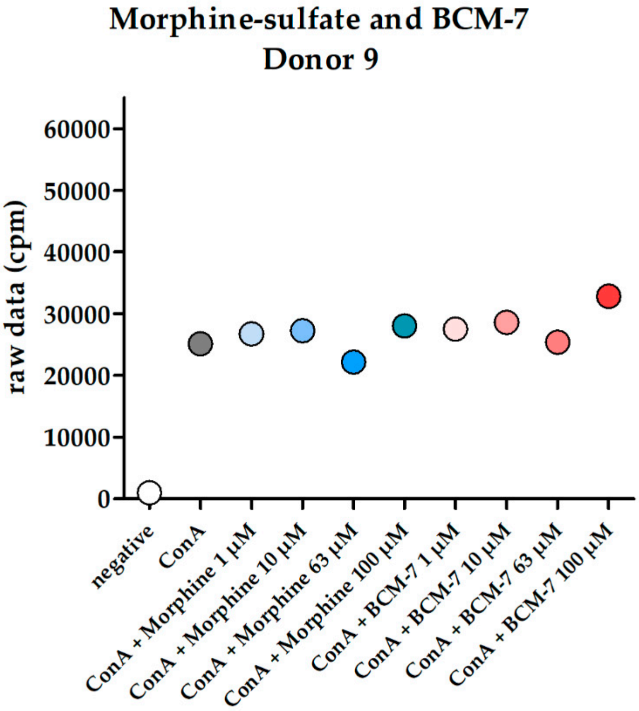

Supplementary Figure S19: Effects of morphine-sulfate and BCM-7 on ConA-stimulated human PBMC of donor 9. Scatter plots represent raw cpm-values of ConA-stimulated PBMC following incubation in morphine-sulfate (from light to dark blue) and BCM-7 (from light to dark red) in various concentrations. ConA-stimulated PBMC served as control (grey). PBMC incubated only in medium (negative control) served as the control for ConA-induced proliferation (white).

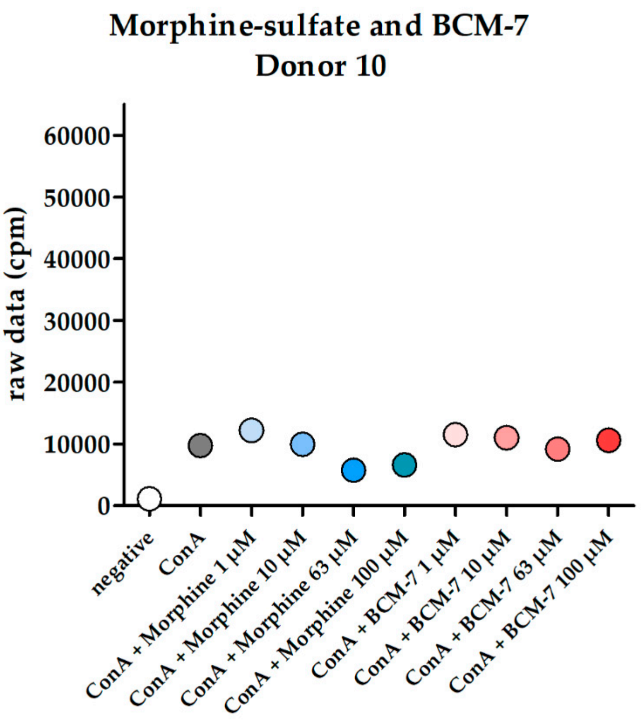

Supplementary Figure S20: Effects of morphine-sulfate and BCM-7 on ConA-stimulated human PBMC of donor 10. Scatter plots represent raw cpm-values of ConA-stimulated PBMC following incubation in morphine-sulfate (from light to dark blue) and BCM-7 (from light to dark red) in various concentrations. ConA-stimulated PBMC served as control (grey). PBMC incubated only in medium (negative control) served as the control for ConA-induced proliferation (white).

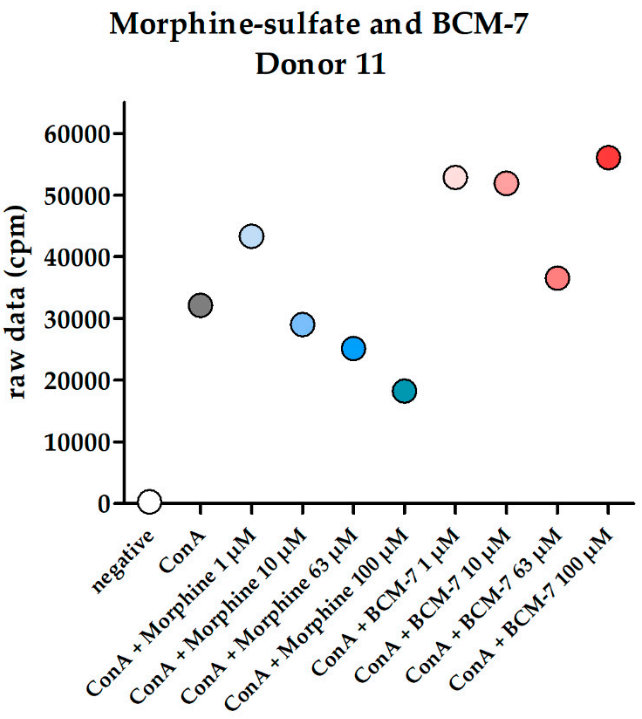

Supplementary Figure S21: Effects of morphine-sulfate and BCM-7 on ConA-stimulated human PBMC of donor 11. Scatter plots represent raw cpm-values of ConA-stimulated PBMC following incubation in morphine-sulfate (from light to dark blue) and BCM-7 (from light to dark red) in various concentrations. ConA-stimulated PBMC served as control (grey). PBMC incubated only in medium (negative control) served as the control for ConA-induced proliferation (white).

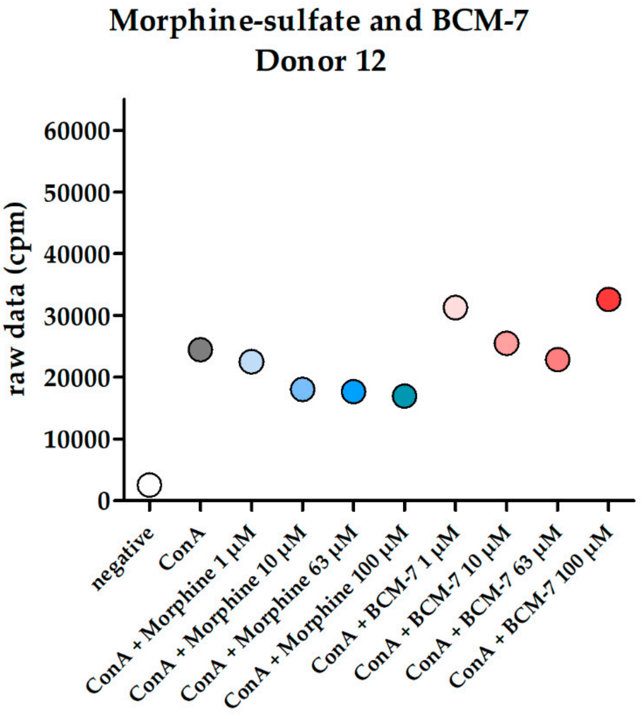

Supplementary Figure S22: Effects of morphine-sulfate and BCM-7 on ConA-stimulated human PBMC of donor 12. Scatter plots represent raw cpm-values of ConA-stimulated PBMC following incubation in morphine-sulfate (from light to dark blue) and BCM-7 (from light to dark red) in various concentrations. ConA-stimulated PBMC served as control (grey). PBMC incubated only in medium (negative control) served as the control for ConA-induced proliferation (white).

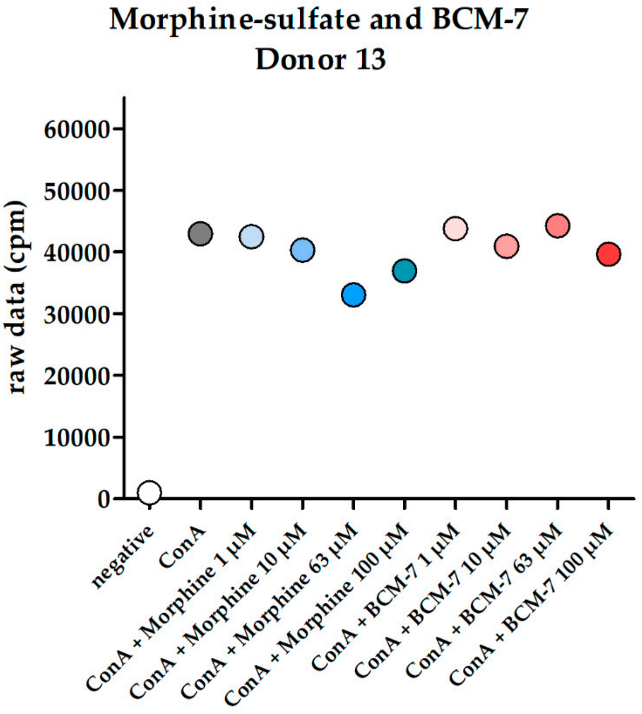

Supplementary Figure S23: Effects of morphine-sulfate and BCM-7 on ConA-stimulated human PBMC of donor 13. Scatter plots represent raw cpm-values of ConA-stimulated PBMC following incubation in morphine-sulfate (from light to dark blue) and BCM-7 (from light to dark red) in various concentrations. ConA-stimulated PBMC served as control (grey). PBMC incubated only in medium (negative control) served as the control for ConA-induced proliferation (white).

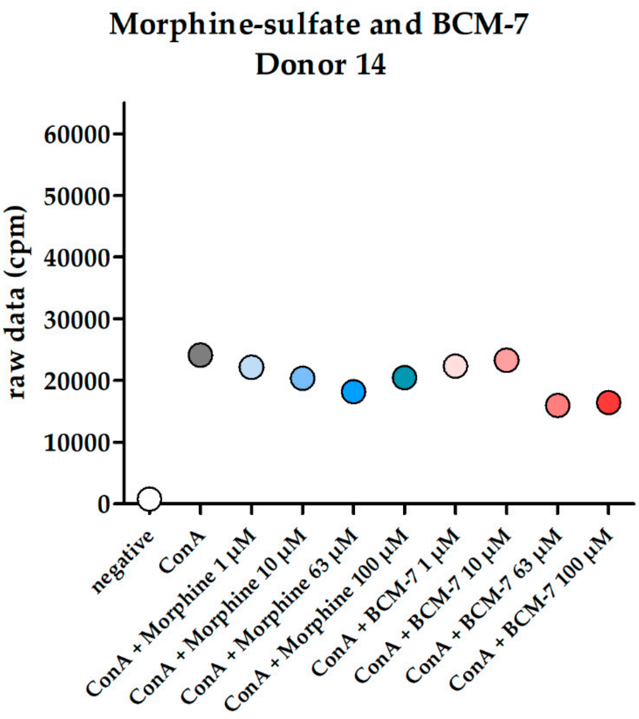

Supplementary Figure S24: Effects of morphine-sulfate and BCM-7 on ConA-stimulated human PBMC of donor 14. Scatter plots represent raw cpm-values of ConA-stimulated PBMC following incubation in morphine-sulfate (from light to dark blue) and BCM-7 (from light to dark red) in various concentrations. ConA-stimulated PBMC served as control (grey). PBMC incubated only in medium (negative control) served as the control for ConA-induced proliferation (white).

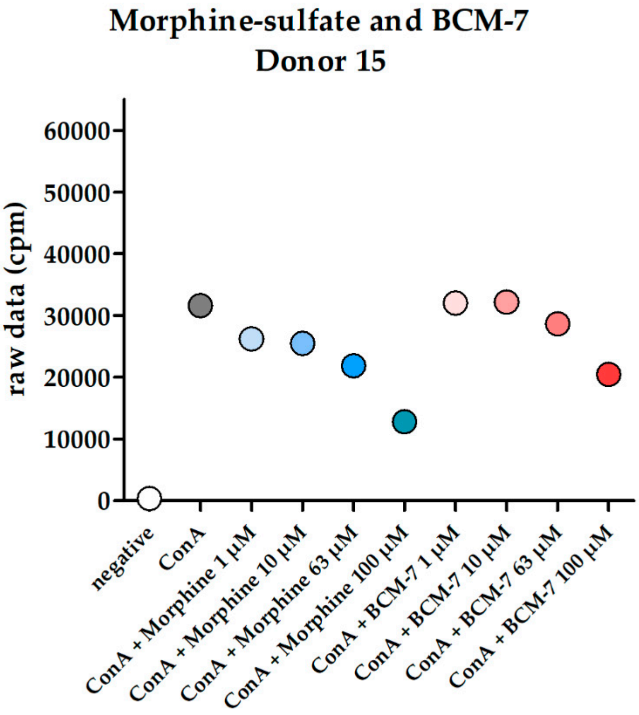

Supplementary Figure S25: Effects of morphine-sulfate and BCM-7 on ConA-stimulated human PBMC of donor 15. Scatter plots represent raw cpm-values of ConA-stimulated PBMC following incubation in morphine-sulfate (from light to dark blue) and BCM-7 (from light to dark red) in various concentrations. ConA-stimulated PBMC served as control (grey). PBMC incubated only in medium (negative control) served as the control for ConA-induced proliferation (white).

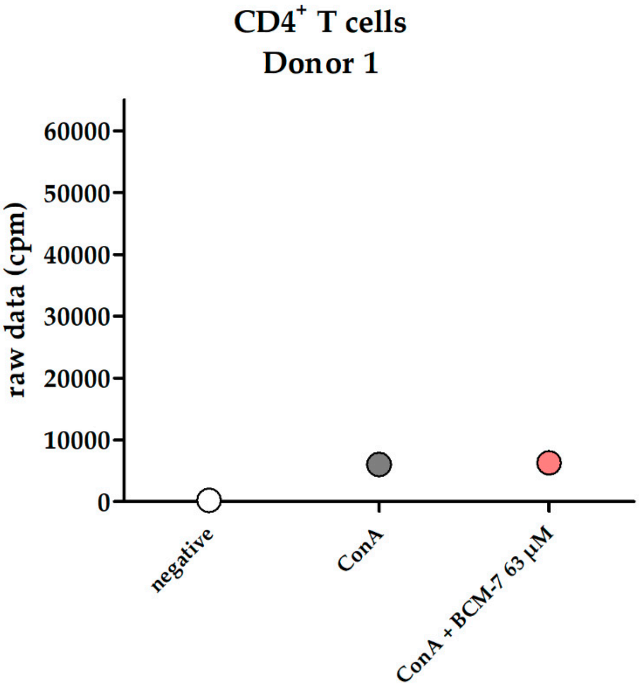

Supplementary Figure S26: Effect of BCM-7 on ConA-stimulated CD4+ T cells of donor 1. Scatter plot represents raw cpm-values of ConA-stimulated CD4+ T cells following incubation in BCM-7 (light red). ConA-stimulated CD4+ T cells served as control (grey). CD4+ T cells incubated only in medium (negative control for CD4+ T cells) served as the control for ConA-induced proliferation (white).

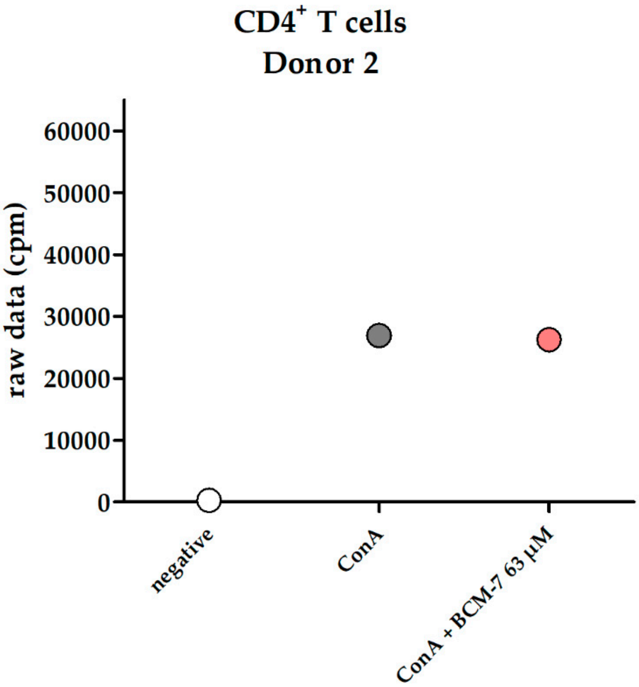

Supplementary Figure S27: Effect of BCM-7 on ConA-stimulated CD4+ T cells of donor 2. Scatter plot represents raw cpm-values of ConA-stimulated CD4+ T cells following incubation in BCM-7 (light red). ConA-stimulated CD4+ T cells served as control (grey). CD4+ T cells incubated only in medium (negative control for CD4+ T cells) served as the control for ConA-induced proliferation (white).

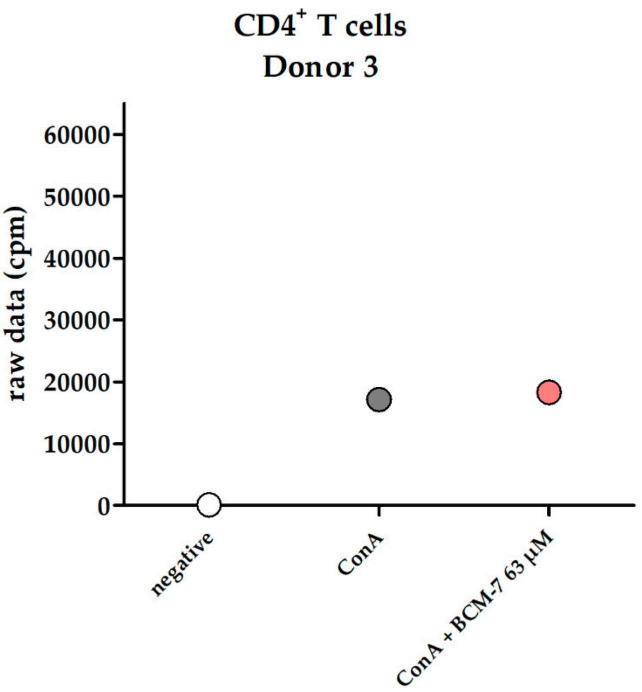

Supplementary Figure S28: Effect of BCM-7 on ConA-stimulated CD4+ T cells of donor 3. Scatter plot represents raw cpm-values of ConA-stimulated CD4+ T cells following incubation in BCM-7 (light red). ConA-stimulated CD4+ T cells served as control (grey). CD4+ T cells incubated only in medium (negative control for CD4+ T cells) served as the control for ConA-induced proliferation (white).

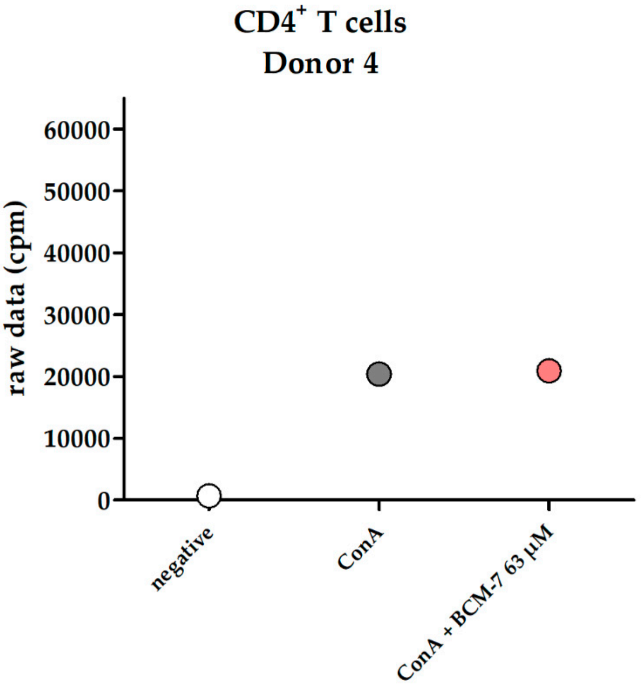

Supplementary Figure S29: Effect of BCM-7 on ConA-stimulated CD4+ T cells of donor 4. Scatter plot represents raw cpm-values of ConA-stimulated CD4+ T cells following incubation in BCM-7 (light red). ConA-stimulated CD4+ T cells served as control (grey). CD4+ T cells incubated only in medium (negative control for CD4+ T cells) served as the control for ConA-induced proliferation (white).

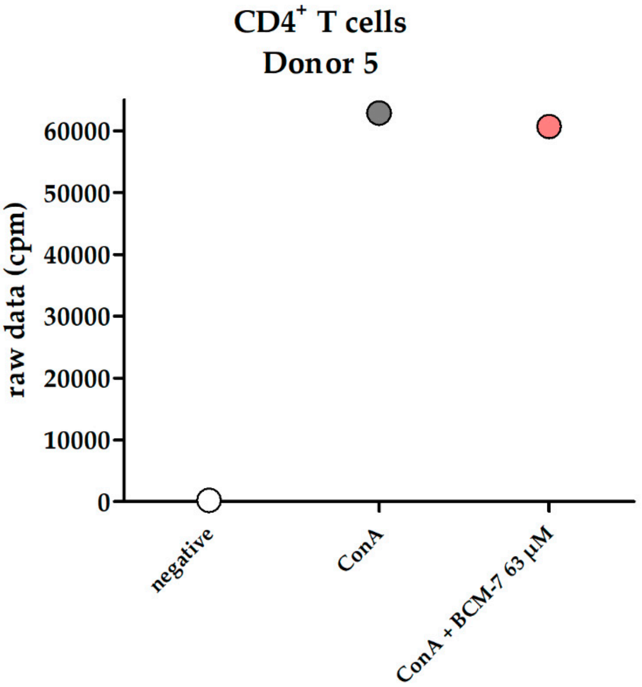

Supplementary Figure S30: Effect of BCM-7 on ConA-stimulated CD4+ T cells of donor 5. Scatter plot represents raw cpm-values of ConA-stimulated CD4+ T cells following incubation in BCM-7 (light red). ConA-stimulated CD4+ T cells served as control (grey). CD4+ T cells incubated only in medium (negative control for CD4+ T cells) served as the control for ConA-induced proliferation (white).

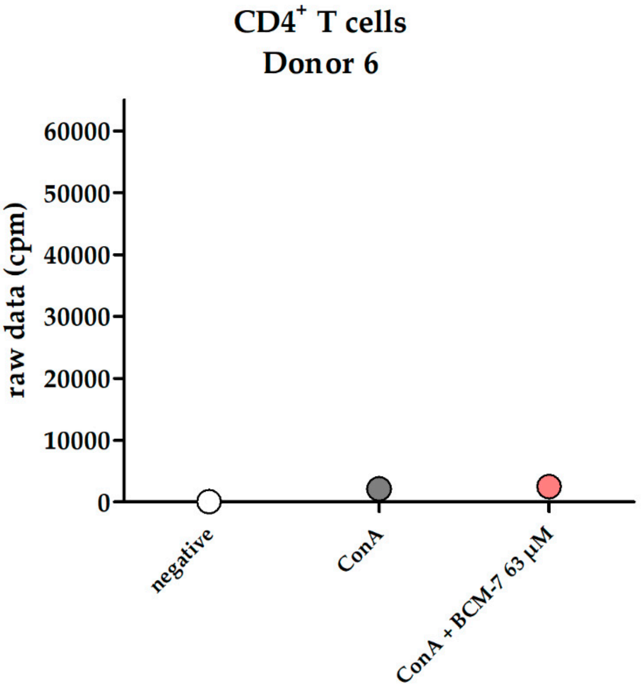

Supplementary Figure S31: Effect of BCM-7 on ConA-stimulated CD4+ T cells of donor 6. Scatter plot represents raw cpm-values of ConA-stimulated CD4+ T cells following incubation in BCM-7 (light red). ConA-stimulated CD4+ T cells served as control (grey). CD4+ T cells incubated only in medium (negative control for CD4+ T cells) served as the control for ConA-induced proliferation (white).

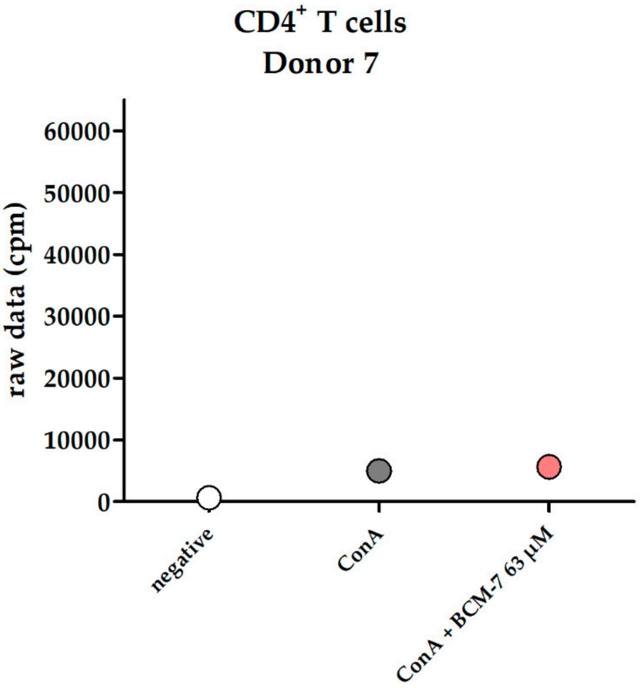

Supplementary Figure S32: Effect of BCM-7 on ConA-stimulated CD4+ T cells of donor 7. Scatter plot represents raw cpm-values of ConA-stimulated CD4+ T cells following incubation in BCM-7 (light red). ConA-stimulated CD4+ T cells served as control (grey). CD4+ T cells incubated only in medium (negative control for CD4+ T cells) served as the control for ConA-induced proliferation (white).

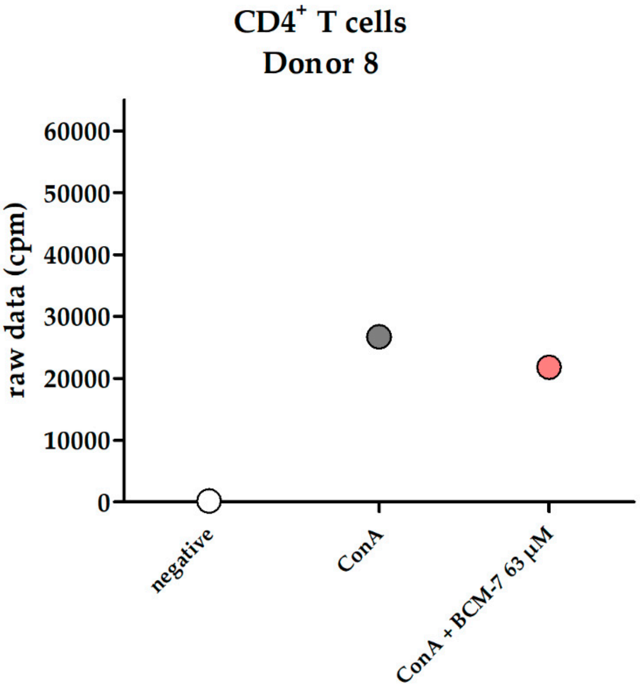

Supplementary Figure S33: Effect of BCM-7 on ConA-stimulated CD4+ T cells of donor 8. Scatter plot represents raw cpm-values of ConA-stimulated CD4+ T cells following incubation in BCM-7 (light red). ConA-stimulated CD4+ T cells served as control (grey). CD4+ T cells incubated only in medium (negative control for CD4+ T cells) served as the control for ConA-induced proliferation (white).

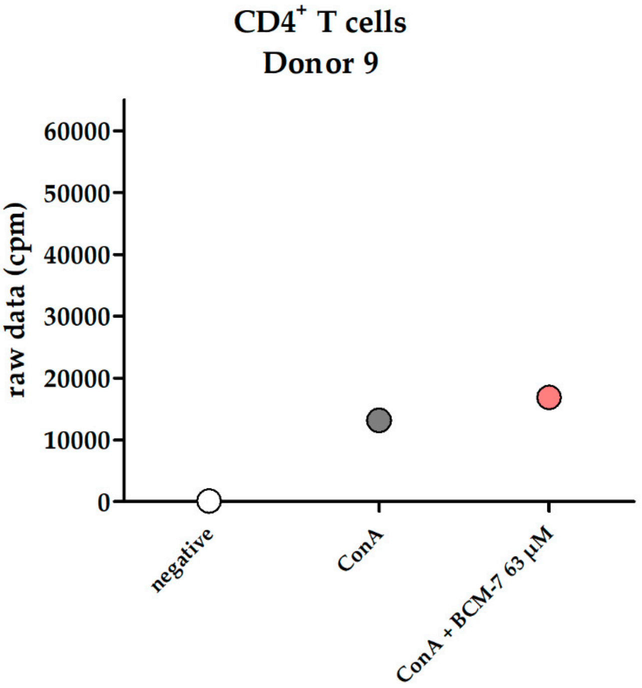

Supplementary Figure S34: Effect of BCM-7 on ConA-stimulated $CD4^+$ T cells of donor 9. Scatter plot represents raw cpm-values of ConA-stimulated $CD4^+$ T cells following incubation in BCM-7 (light red). ConA-stimulated $CD4^+$ T cells served as control (grey). $CD4^+$ T cells incubated only in medium (negative control for $CD4^+$ T cells) served as the control for ConA-induced proliferation (white).

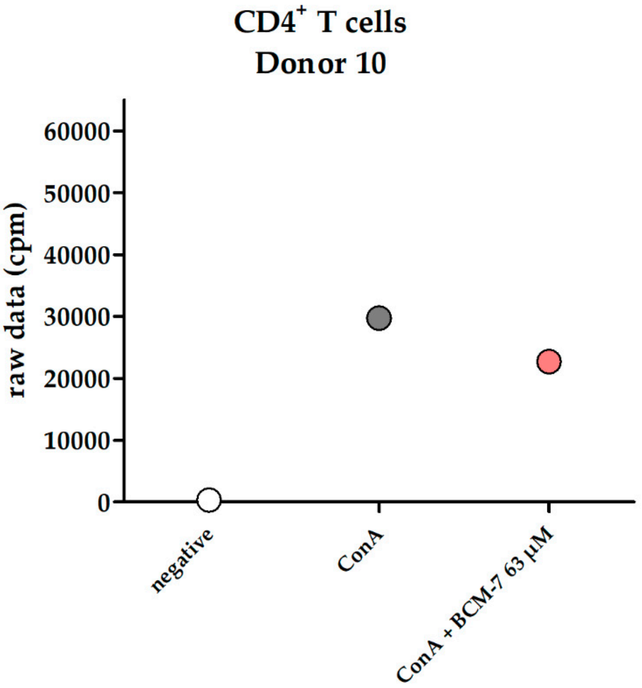

Supplementary Figure S35: Effect of BCM-7 on ConA-stimulated $CD4^+$ T cells of donor 10. Scatter plot represents raw cpm-values of ConA-stimulated $CD4^+$ T cells following incubation in BCM-7 (light red). ConA-stimulated $CD4^+$ T cells served as control (grey). $CD4^+$ T cells incubated only in medium (negative control for $CD4^+$ T cells) served as the control for ConA-induced proliferation (white).

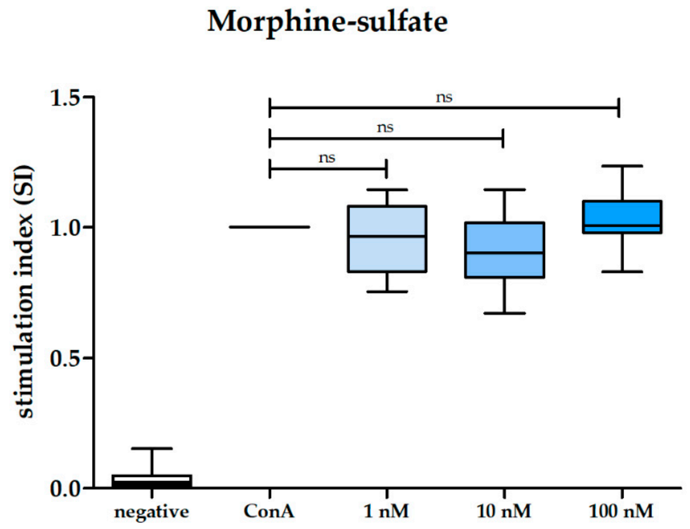

Supplementary Figure S36. Effect of morphine-sulfate on ConA-stimulated PBMC. Box and whiskers plots represent mean stimulation indices of ConA-stimulated PBMC following incubation in morphine-sulfate in various concentrations (from light to medium blue), n = 15. ConA-stimulated PBMC served as control and were set to 1 (black), n = 15. PBMC incubated only in medium (negative control) served as the control for ConA-induced proliferation (white), n = 15. Non-parametric Kruskal-Wallis test followed by Dunn's post hoc test was performed to test for statistical significance; ns, $p \geq 0.05$.

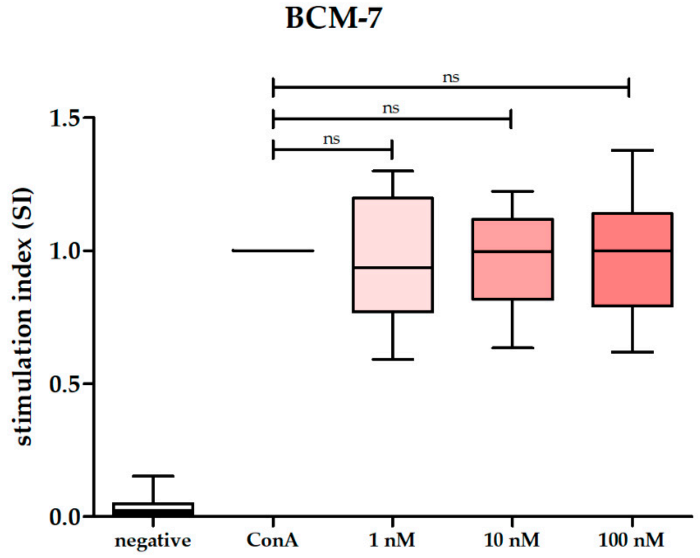

Supplementary Figure S37. Effect of BCM-7 on ConA-stimulated PBMC. Box and whiskers plots represent mean stimulation indices of ConA-stimulated PBMC following incubation in BCM-7 in various concentrations (from light to medium red), n = 15. ConA-stimulated PBMC served as control and were set to 1 (black), n = 15. PBMC incubated only in medium (negative control) served as the control for ConA-induced proliferation (white), n = 15. Non-parametric Kruskal-Wallis test followed by Dunn's post hoc test was performed to test for statistical significance; ns, $p \geq 0.05$.
